# Structural insights into a bacterial terpene cyclase fused with haloacid Dehalogenase-like phosphatase

**DOI:** 10.1039/d5sc04719f

**Published:** 2025-07-28

**Authors:** Keisuke Fujiyama, Hiroshi Takagi, Nhu Ngoc Quynh Vo, Naoko Morita, Toshihiko Nogawa, Shunji Takahashi

**Affiliations:** a Natural Product Biosynthesis Research Unit, RIKEN Center for Sustainable Resource Science Wako Saitama 351-0198 Japan shunjitaka@riken.jp; b Molecular Structure Characterization Unit, RIKEN Center for Sustainable Research Science Wako Saitama 351-0198 Japan

## Abstract

Terpene cyclases (TCs), consisting of various combinations of α, β, and γ domains, have been extensively studied. Recently, non-canonical enzymes comprising a TCβ domain and a haloacid dehalogenase (HAD)-like domain (referred to as HAD-TCβ) have been discovered. However, their overall structure remains unclear. In this study, we determined the co-crystal structures of drimenol synthase from *Aquimarina spongiae* (AsDMS), which catalyzes the conversion of farnesyl pyrophosphate (1) into drimenol (2). Crystallographic analyses of the enzyme bound to substrates 1 and drimenyl monophosphate (3) demonstrated that the TCβ domain catalyzes a class II cyclization reaction initiated by protonation, whereas the HAD domain catalyzes a phosphatase-like dephosphorylation reaction dependent on a divalent metal. Crystallographic and gel filtration analyses revealed that AsDMS adopts a dimeric assembly. This dimerization positioned the TCβ and HAD domains to facilitate efficient substrate transfer *via* electrostatic substrate channeling. Furthermore, to investigate the structure–function relationship of the AsDMS TCβ domain, we used AlphaFold2 to model the structure of the fungal albicanol (4) synthase. Comparative analysis of active-site residues between AsDMS and fungal 4-synthase enabled rational protein engineering, converting AsDMS activity from 2-synthase to 4-synthase. This study provides insights into the biosynthesis of valuable drimane-type sesquiterpenes *via* targeted mutagenesis.

## Abstract 2

This study aimed to elucidate the function of the bacterial bifunctional enzyme AsDMS, which catalyzes the conversion of farnesyl pyrophosphate into drimenol. Structural, mutational and biochemical analyses revealed the molecular mechanisms of AsDMS.

## Graphical abstract

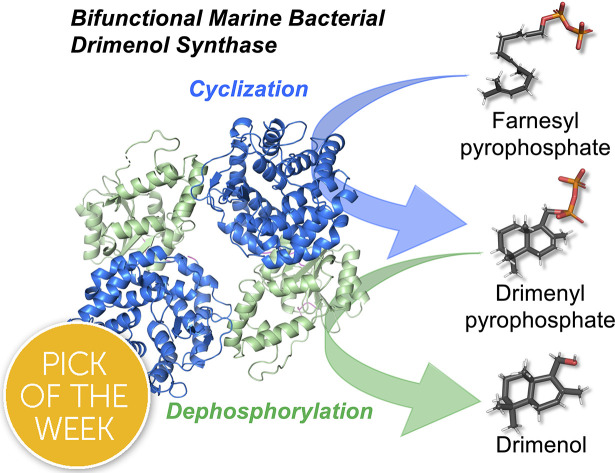

## Introduction

Terpenoids constitute one of the largest classes of natural products and are widely used in fragrances, perfumes, pharmaceuticals, pesticides, and repellents.^1–4^ Terpenoids are broadly distributed in nature and produced by animals, plants, fungi, bacteria, protists, and viruses.^5–9^ Recently, marine organisms such as marine bacteria, soft corals, and sponges have been identified as sources of unique and structurally complex terpenoids,^10–13^ potentially offering promising avenues for addressing or overcoming challenging and emerging infectious diseases. The diverse biological activities of terpenoids are attributed to their structural diversity, which is strictly governed by terpenoid biosynthetic enzymes.

Terpenoid biosynthesis begins with two precursors: isopentenyl pyrophosphate and dimethylallyl pyrophosphate. These precursors undergo condensation reactions, leading to the formation of geranyl pyrophosphate, farnesyl pyrophosphate (1), and geranylgeranyl pyrophosphate.^14,15^ Subsequently, terpene cyclases (TCs) catalyze the formation of diverse carbon skeletons, followed by further modifications (*e.g.*, dephosphorylation,^16,17^ oxidation,^17^ reduction,^18^ methylation,^19^ glycosylation,^20,21^ and acetylation^16^) to yield bioactive terpenoid compounds.^7,22^ Regio- and stereoselective cyclization reactions catalyzed by TCs play crucial roles in determining terpene skeletons and their recognition by modifying enzymes. Therefore, considerable research efforts have been dedicated to elucidating the molecular mechanisms of TC enzymes.^23–26^

In general, canonical TCs consist of α, β, and γ domains, or combinations thereof. To date, various combinations, including α, β, α/β, β/γ, and α/β/γ domains, have been identified.^15,26,27^ Certain TCs with unique domain assemblies have evolved independently to achieve bifunctionality. For instance, fusicoccadiene synthase, which is involved in fusicoccin A biosynthesis, possesses prenyltransferase and diterpene cyclase domains, enabling it to independently catalyze isoprene condensation and subsequent cyclization.^21^ Fusicoccadiene synthase efficiently synthesizes fusicoccadienes by physically combining the catalytic sites of the two domains *via* a flexible linker region.^28^

**Fig. 1:**
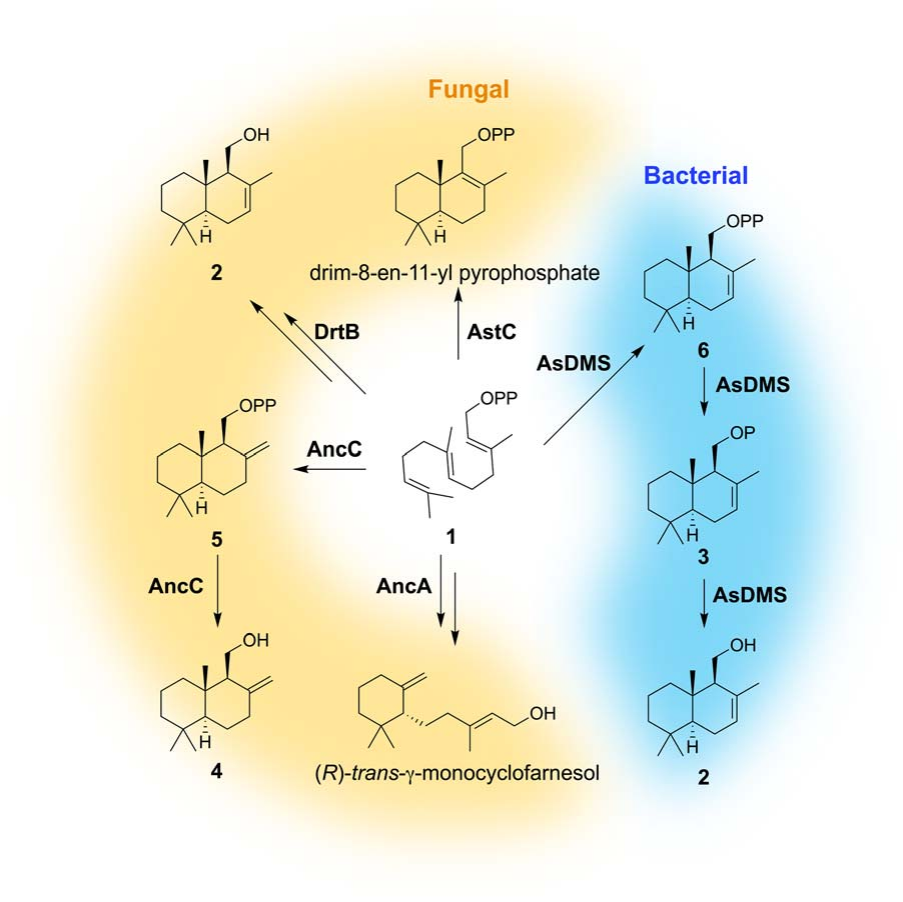
Drimane-type sesquiterpenes produced by fungal and bacterial HAD-TCβ enzymes. HAD-TCβ is a bifunctional enzyme that catalyzes cyclization and subsequent dephosphorylation.^16,17,29,30^

As another example, we have previously reported AstC, a fusion enzyme consisting of a TCβ domain and a haloacid dehalogenase (HAD) domain (HAD-TCβ), during the study of the biosynthetic mechanism of astellolides.^16^ Subsequently, HAD-TCβ enzymes have been identified in various bacteria and fungi, catalyzing the conversion of 1 into sesquiterpene alcohols possessing a drimane skeleton (Fig. 1).^17,29,30^ Moreover, the marine bacterial HAD-TCβ drimenol (2) synthase (AsDMS), derived from *Aquimarina spongiae*, has been demonstrated to exhibit superior catalytic efficiency compared with *Valeriana officinalis* DMS, a typical plant-derived TC.^30,31^ Additionally, the precursor scaffolds produced by fungal HAD-TCβs are of significant importance in the biosynthesis of bioactive natural products.^17^ Enzyme engineering based on structural insights into HAD-TCβs is expected to enable the efficient production of valuable compounds. However, the crystal structures of HAD-TCβ enzymes remain to be experimentally elucidated.

In this study, we report the crystallographic analyses of AsDMS, an enzyme that converts substrate 1 into product 2, and the biochemical characterization of site-specific variants. The obtained crystal structure of AsDMS represents the first experimentally determined structure of a HAD-TCβ enzyme, revealing distinct substrate-binding pockets for the HAD and TCβ domains. The co-crystal structures of AsDMS bound to substrate 1 and drimenyl monophosphate (3) enabled the elucidation of ligand-binding conformations and interactions at the atomic level. Site-directed mutagenesis was performed to assess ligand interactions and identify critical catalytic residues involved in cyclization and dephosphorylation reactions. We demonstrated that pyrophosphate release and metal dependence occurred within the HAD domain of AsDMS. Furthermore, comparative structural analyses of the AsDMS crystal structure and predicted fungal HAD-TCβ structures led to the creation of engineered AsDMS variants, which gained the ability to synthesize albicanol (4). Through crystal structure analysis and sequence comparisons of enzymes with different product selectivities, various variants were constructed, and their enzymatic activities were evaluated. Based on these findings, this study discusses the cyclization and dephosphorylation mechanisms facilitated by this bifunctional enzyme.

## Results and discussion

### The overall structure of AsDMS and assembly of bacterial HAD-TCβs

**Fig. 2:**
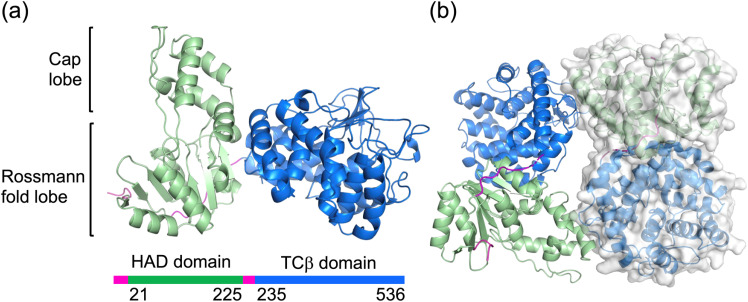
Overall structure of AsDMS. (a) Crystal structure of AsDMS d18/D333N variant. The HAD domain, TCβ domain, and linkers were colored green, blue, and magenta, respectively. (b) The AsDMS dimer structure is depicted as a ribbon (left side) and a surface model (right side).

To prepare an enzyme suitable for the structural determination of AsDMS, we first conducted disorder^32,33^ and secondary structure predictions.^34^ Because wild-type AsDMS possesses a long disordered region at its N-terminus, we truncated 18 residues from the N-terminus to generate AsDMS d18 (Fig. S1). Additionally, to obtain a co-crystal structure with substrate 1, we introduced the D333N mutation^30^ into AsDMS d18 (AsDMS d18/D333N), which abolished the protonation-initiated cyclization reaction. We determined the crystal structure of AsDMS d18/D333N at 2.30 Å resolution. The crystal structure revealed that AsDMS consists of N-terminal HAD and C-terminal TCβ domains connected by a highly flexible, disordered linker (Fig. 2). The HAD domain comprised a Cap lobe and a Rossmann fold lobe.

The asymmetric unit of the crystal structure contained two AsDMS molecules. The evolutionary protein–protein interface classifier (EPPIC) analyses,^35–39^ which can predict the assemblies and interfaces of protein crystals, estimated the biological relevance scores of the oligomerization states, assigning a score of 1.00 for a monomer with a *C*_1_ symmetry axis and 0.55 for a dimer with a *C*_2_ symmetry axis. Gel filtration analysis was conducted to evaluate the oligomeric state of AsDMS and confirmed that the major peak corresponded to the dimer (Fig. S2). These results suggested that AsDMS forms a dimeric structure with a *C*_2_ symmetry axis. Given the absence of a universally accepted biochemical method for definitively determining oligomeric states, we sought to further investigate the oligomerization dynamics of AsDMS homologs. To this end, we performed gel filtration analyses on other marine bacterial HAD-TCβ enzymes, including *Flavivirga eckloniae* ECD14^T^ DMS (FeDMS) and *Aquimarina* sp. AU119 DMS (A119DMS).^30^ These extended analyses revealed that the dimeric state is predominant in these DMS enzymes as well, suggesting that AsDMS may adopt different quaternary structures depending on the environmental context (Fig. S2).

To further investigate the domain assembly of AsDMS revealed by its crystal structure, structural similarity searches were performed using the PDBeFold server.^40,41^ No proteins structurally similar to the entire AsDMS molecule were identified; however, when searches were conducted using individual HAD and TCβ domains, structurally similar proteins were obtained. Among the top 20 identified proteins, only AsDMS exhibited structural differences within the core region of the Rossmann fold, notably lacking the conventional C-terminal α-helix involved in typical folding and instead possessing an additional N-terminal α-helix (Fig. S3). In contrast, the TCβ domain of AsDMS showed structural similarity with several proteins (Fig. S4a), five of which are involved in terpenoid biosynthesis (Fig. S4b). Among these, the core α-helical bundle structure was highly conserved, with merosterolic acid synthase^27^ showing the highest similarity (Fig. S4c). It is proposed that AsDMS originated through the fusion of a HAD-like phosphatase domain and a standalone TCβ domain enzyme, potentially undergoing structural optimization during molecular evolution, leading to changes in the Rossmann fold of the HAD domain. Additionally, comparative analysis between the crystal structure of AsDMS and AlphaFold2 (ref. 42)-predicted structures of fungal HAD-TCβ enzymes revealed that the fungal HAD domains^16,17,29^ retained the typical Rossmann fold characteristic of common HAD enzymes (Fig. S5). Although there are currently no reports on the oligomeric state of functionally characterized fungal HAD-TCβs, these findings suggest potential structural and oligomeric differences between fungal and bacterial HAD-TCβ enzymes.

### The co-crystal structure of 1-bound AsDMS and mutagenesis study in TCβ domain

**Fig. 3:**
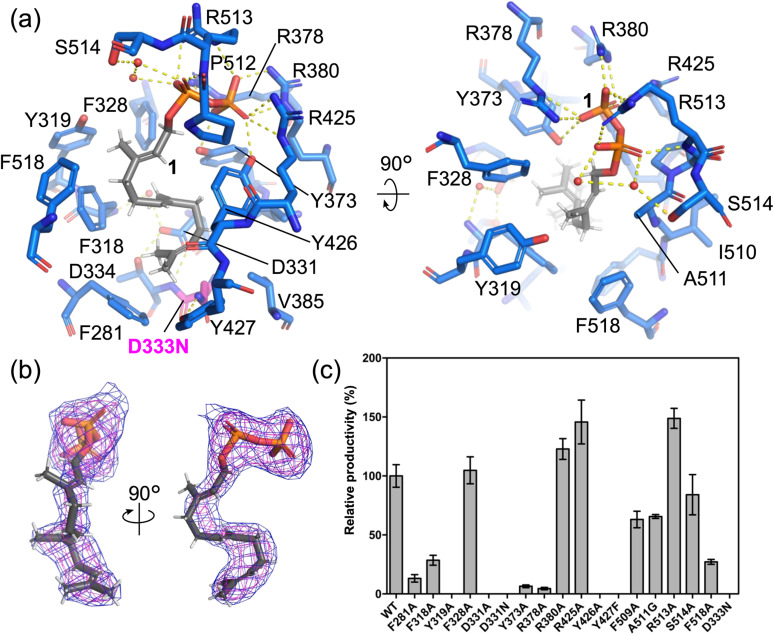
Structures in the TCβ domain of 1-bound AsDMS. (a) Binding conformation of 1 in the TCβ domain. The yellow dashed lines indicate hydrogen bonds (within 3.5 Å). The red sphere models are water molecules. D333N is colored magenta. (b) The polder map^45^ of 1 in TCβ domain. The magenta and blue mesh indicate the polder map of 1 contoured at 3.0 and 2.0 *σ*, respectively. (c) Enzyme activity of TCβ domain variants was obtained as the mean ± standard deviation (SD) of three independent experiments (Fig. S7). Relative productivity was calculated based on the specific activity of wild-type AsDMS. WT means wild-type AsMDS.

To elucidate the cyclization mechanism catalyzed by the TCβ domain of AsDMS, we determined the crystal structure of substrate 1-bound AsDMS d18/D333N at a resolution of 2.60 Å (Fig. 3a). The electron density map of 1 was clearly observed at the center of the TCβ domain (Fig. 3b). The binding mode markedly differs from that of the class II TC *Streptomyces showdoensis* DMS,^43^ which requires Mg^2+^ for phosphate binding. Instead, it resembled merosterolic acid synthase,^27^ a monodomain class II cyclase that specifically recognizes the hydrophilic head of its substrate (Fig. S6).

Based on the co-crystal structure of the TCβ domain, site-directed mutations were introduced to elucidate the role of each amino acid residue forming the pocket that binds substrate 1, and the relative productivities of the variants were compared. Regarding the binding conformation of the pyrophosphate moiety, 1 formed salt bridges with the residues R378, R380, R425, and R513. 1 also formed hydrogen bonds with Y373 and Y426, and interacted with S514 through water-mediated hydrogen bonds (Fig. 3a). The Y373A and Y426A variants completely lost their enzymatic activity and R378A lost its activity of 96%, indicating that these residues are critical for phosphate recognition. The R380A, R425A, and R513A variants showed increased relative productivities of 22%, 45%, and 48%, respectively (Fig. S7), suggesting that these alterations may optimize the binding mode of substrate 1 or facilitate product release since the arginine residues were not completely conserved among the other DMSs (Fig. S8 and S9). The S514A variant reduced 17% of its enzymatic activity, suggesting that indirect water-mediated hydrogen bonding was not essential. The hydrophobic tail of 1 adopts a binding conformation stabilized by interactions with multiple residues, grouped by their proposed functional roles: catalytic residues (D331, D333, and Y427), residues involved in π–cation interactions (F281, F318, F328, F509, and F518), and residues involved in van der Waals interactions (Y319, A511, and P512) (Fig. 3a). Similarly, we performed mutagenesis of the listed amino acids and assessed their activities. The catalytic residue variants (D331A, D331N, D333N,^30^ and Y427F) exhibited a complete loss of enzymatic activity, supporting the idea that the D333 residue, which forms a hydrogen bond with the Y427 residue, catalyzes protonation at the C10 position of 1 (Fig. 3a). The π–cation interaction variants F281A, F318A, and F518A exhibited reduced enzymatic activity of 87%, 37%, and 73%, respectively, suggesting that these residues play an important role in stabilizing carbocation intermediates by binding to substrate 1 during cyclization, or both.^24^ The residue F518 was located near the C4 position of 1, suggesting that the C–H⋯π interaction between the carbocation intermediate and the aromatic ring of F518 determines the product selectivity (Fig. S10 and Scheme S1).^44^ However, F518 is not appropriate for proton abstraction, and Y319 is clearly distant from the C4 carbocation. In addition, the backbone carbonyl group of L510 didn't face the C4 position of 1. Therefore, a water molecule might abstract the proton and thereby terminate the cyclization. Contrary to our expectations, the variants F328A showed increased 4% of enzymatic activity and F509A showed reduced 37.3% of enzymatic activity, indicating that π–cation interaction at F328 and F509 is not critical. The van der Waals interaction variant Y319A completely lost its activity, suggesting that the Y319 mutation, located near key residues (F318, F328, and F518), disrupted the catalytic pocket. A511G activity was lost at 34.4%, suggesting that A511 functions as a pocket-defining residue.

### Alternation of product selectivity of TCβ domain

**Fig. 4:**
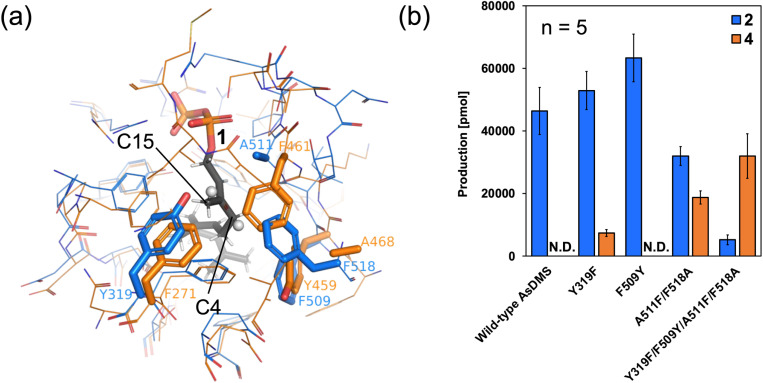
Key residues-switching experiment of AsDMS and AncC. (a) Only the residues that differ in the active site are shown as stick models. White sphere models indicate hydrogen atoms at the C4 and C15 positions of 1. (b) The reaction product profiles of AncC-mimic AsDMS variants. The reaction products were quantified using GC/MS. Data are presented as means ± standard deviation from five replicates. N.D. indicates ‘not detected’.

To elucidate the key residues determining product selectivity in HAD-TCβ enzymes, we performed comparative analyses using the fungal 4-synthase AncC.^17^ Structural comparison between the substrate 1-bound AsDMS crystal structure and the fungal AncC revealed that substrate-interacting residues within their TCβ domains were highly conserved, with differences observed only at four residues. Specifically, residues Y319, F509, A511, and F518 in AsDMS corresponded to residues F271, Y459, F461, and A468 in AncC, respectively (Fig. 4a). Therefore, we prepared AsDMS variants in which the amino acid residues were substituted with those corresponding to AncC and analyzed their product profiles using gas chromatography-mass spectrometry. The double variant (A511F/F518A) produced compounds 2 and 4, moreover the quadruple variant (Y319F/F509Y/A511F/F518A) primarily produced compound 4 (Fig. 4b). Because the product selectivity of TCs is known to be modulated by C–H⋯π interactions between carbocation intermediates and phenyl groups,^44^ the phenyl group introduced by the A511F/F518A mutation is considered to critically influence product selectivity through a C–H⋯π interaction with the C15 position of the carbocation intermediate, thereby promoting the formation of an *exo*-methylene-type drimane skeleton (Fig. 4a and Scheme S2). The Y319F variant also produced compounds 2 and 4, suggesting the potential to shift the resonance form of the intermediate. The F509Y variant increased the production of 2 but 4 was not detected. The 1 binding pocket and variant analyses suggested that the Y319F and F509Y mutations contributed to the formation of a substrate-binding pocket favorable for 4 production.

### Isolation of drimenyl phosphate intermediate and characterization of enzyme activity

In the fungal HAD-TCβ enzyme AncC, production of 4 from 1 proceeds through the cyclized intermediate albicanyl pyrophosphate (5), with subsequent dephosphorylation of the pyrophosphate moiety catalyzed by the HAD domain (Fig. 1).^17^ To elucidate the reaction mechanism involving the HAD domain in AsDMS, we isolated a cyclized intermediate. Given that typical HAD-like phosphatases exhibit divalent metal-ion dependency,^46,47^ a TCβ domain reaction was conducted in the presence of ethylenediaminetetraacetic acid (EDTA), and a cyclized intermediate bearing a phosphate group was isolated. ^1^H-, ^13^C-, and ^31^P-NMR spectroscopy (Table S3 and Fig. S11), as well as HR-ESI-MS analysis (*m*/*z* 301.1571 [M − H]^−^, calculated mass 301.1569, C_15_H_26_O_4_P) confirmed that the accumulated product is drimenyl monophosphate (3) (Fig. S12). Furthermore, the treatment of 3 with either bacterial alkaline phosphatase or AsDMS D333N resulted in the production of 2 in both cases (Fig. S13). The cyclization reaction catalyzed by the AsDMS TCβ domain is Mg^2+^-independent, whereas the subsequent dephosphorylation step mediated by the HAD domain is Mg^2+^-dependent. AsDMS exhibited enzyme activity in the presence of divalent metal ions other than Mg^2+^. However, this activity was inhibited by Ca^2+^ (Fig. S14). Collectively, these results suggest that the HAD domain of AsDMS shares metal dependency with typical HAD-like phosphatases.^46,48^ Considering the known class II TCβ reaction mechanism of the TCβ domain and previous studies,^16,17,43^ the authentic product of the TCβ domain is presumed to be drimenyl pyrophosphate (6) (see also further experiments below). However, in our experiments, the use of Ca^2+^ to precipitate reaction intermediates (as calcium phosphate) may have led to hydrolysis of the pyrophosphate moiety, resulting in the formation of 3.^49^

### Dephosphorylation mechanisms and co-crystal structure analysis in the HAD domain

**Fig. 5:**
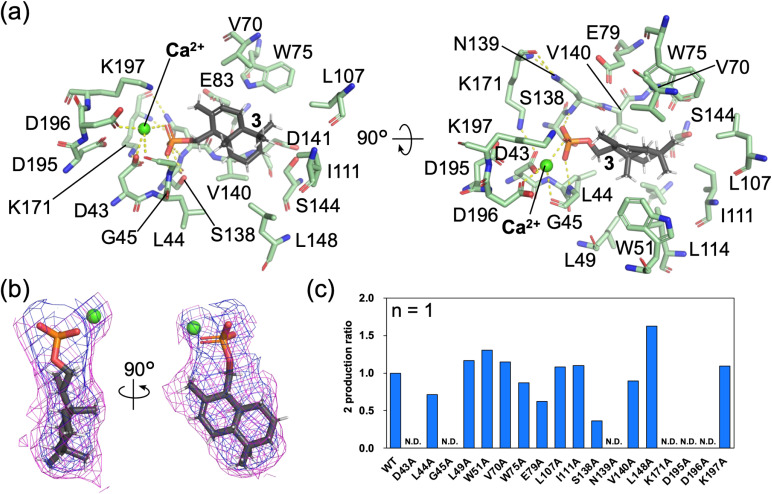
Structures in the HAD domain of 3-bound AsDMS. (a) Binding conformation of 3 in the HAD domain. The yellow dashed lines indicate hydrogen bonds (within 3.5 Å). (b) The polder map^45^ of 3 in the HAD domain. The magenta and blue mesh indicate the polder map of 3 and Ca^2+^ contoured at 3.0 and 2.0 *σ*, respectively. (c) Evaluation of enzyme activity of the HAD domain variants. The product 2 was analyzed using GC/MS. 2 production ratio was calculated based on the specific activity of wild-type AsDMS. WT means wild-type AsMDS. N.D. indicates ‘not detected’.

To clarify the dephosphorylation mechanism mediated by the HAD domain, we performed MESG (2-amino-6-mercapto-7-methylpurine ribonucleoside, 7) assays capable of monitoring inorganic phosphate (Pi) production (Fig. S15).^50,51^ In control experiments with pyrophosphate and pyrophosphatase, Pi production was confirmed to be time-dependent (Fig. S16a and b; line F). When wild-type AsDMS was incubated with 1, Pi was released both in the presence and absence of pyrophosphatase (Fig. S16a and c; lines G and J). When wild-type AsDMS was incubated with 3, the release of inorganic phosphate (Pi) was also observed (Fig. S16a and c; lines H and K). These results collectively suggest that AsDMS releases Pi in a stepwise manner (Scheme S1). As anticipated, when the HAD domain-inactivated variant (AsDMS D43A) (Fig. 5) was incubated with 1, no dephosphorylation was observed (Fig. S16a and d; lines N and O). Additionally, when the TCβ domain-inactivated variant (AsDMS D333N) was incubated with 1, no dephosphorylation reaction was observed (Fig. S16a and d; line M), demonstrating that 1 is not recognized by the HAD domain.

To detect the drimenyl pyrophosphate intermediate, rather than Pi, we also performed enzymatic reactions under HAD domain-inactivated conditions—either using the AsDMS D43A variant or adding EDTA to chelate Mg^2+^. Importantly, HR-ESI-MS analysis revealed a clear peak corresponding to the pyrophosphorylated intermediate (*m*/*z* 381.1229 [M − H]^−^, calculated mass 381.1232, C_15_H_27_O_7_P_2_), while no monophosphorylated intermediate was observed (Fig. S17). Collectively, these findings provided direct evidence that AsDMS initially generates a pyrophosphate intermediate, supporting a stepwise dephosphorylation mechanism by the HAD domain that converts 6 into 2*via*3 (Scheme S1).

Given that the isolated compound 3 has been experimentally verified as an intermediate substrate for AsDMS, kinetic analyses were conducted. The wild-type HAD domain of AsDMS exhibited a *K*_m_ value of 5.81 ± 0.81 μM and a *k*_cat_ value of 0.559 ± 0.020 s^−1^ with a *k*_cat_/*K*_m_ value of 0.096 s^−1^ μM^−1^ for 3 (Fig. S18). The specificity constant of 3 was significantly higher than that of 1 (*K*_m_ = 9.59 ± 2.15 μM, *k*_cat_ = 0.086 ± 0.01 s^−1^, *k*_cat_/*K*_m_ = 0.0090 s^−1^ μM^−1^),^30^ suggesting that the reaction catalyzed by TCβ domain is the rate limiting step in the AsDMS reaction.

To elucidate the dephosphorylation mechanism of 3 catalyzed by the HAD domain, we determined the crystal structure of AsDMS d18/D333N bound to 3 at a resolution of 2.90 Å, in the presence of Ca^2+^, an inhibitor of the HAD domain^48^ (Fig. 5a). An electron density map of 3 was observed within the HAD domain (Fig. 5b). The phosphate moiety of 3 formed hydrogen bonds with the side chain of residue S138 as well as with the main-chain atoms of residues G45 and N139 (Fig. 5a). Ca^2+^ coordinated with residues D43, D195, and D196, the main-chain carbonyl group of G45, and the phosphate moiety of 3. Residue D43 directly interacted with Ca^2+^, orienting its carbonyl group toward the phosphorus atom of the phosphate group. Thus, residue D43 is proposed to serve as a catalytic residue that mediates dephosphorylation. Furthermore, considering that residues D195 and D196 constitute the metal-binding motif.

To verify the functions of active-site residues in the HAD domain, we prepared various variants (D43A, L44A, G45A, L49A, W51A, V70A, W75A, E79A, L107A, I111A, S138A, N139A, V140A, L148A, K171A, D195A, D196A, and K197A) and evaluated their dephosphorylation activities using 3 as the substrate. Variants D43A, G45A, N139A, K171A, D195A, and D196A exhibited a complete loss of enzymatic activity (Fig. 5c). These results indicate that D43 and K171 residues serve as catalytic residues for dephosphorylation of 3 by the HAD domain, while residues D195 and D196 are essential for Mg^2+^ binding.^30,52,53^ The carbonyl group of G45 was a coordinating with the metal, also indicating that also G45 is an important ligand for a Mg^2+^ binding. The complete loss of activity observed for the N139A variant likely resulted from the structural disruption of the substrate-binding pocket. Moreover, as previous studies have reported that Mg^2+^ and conserved Ser/Thr residues in HAD enzymes are crucial for phosphate binding,^46,47,54^ the observed reduction in enzymatic activity of variant S138A is presumably due to disruption of interactions with the phosphate moiety of 3.

Furthermore, to predict the binding mode of initial pyrophosphate substrate 6, docking simulations were performed based on the crystal structure of 3-bound AsDMS as a template. The results revealed additional interactions with N139, K197, and N200 at the distal phosphate group, whereas the rest of the binding conformation closely resembled that of 3 (Fig. S19). Thus, the dephosphorylation of 6 was considered to proceed in a manner similar to that of 3. Additionally, 3 is recognized as a substrate by the HAD domain, and our previous study showed that the K197D variant retains its activity, suggesting that the interactions of only the outer phosphate moiety with N139, K197, and N200 might support binding, but are not considered essential for substrate recognition.

### Catalytic mechanism of AsDMS

**Fig. 6:**
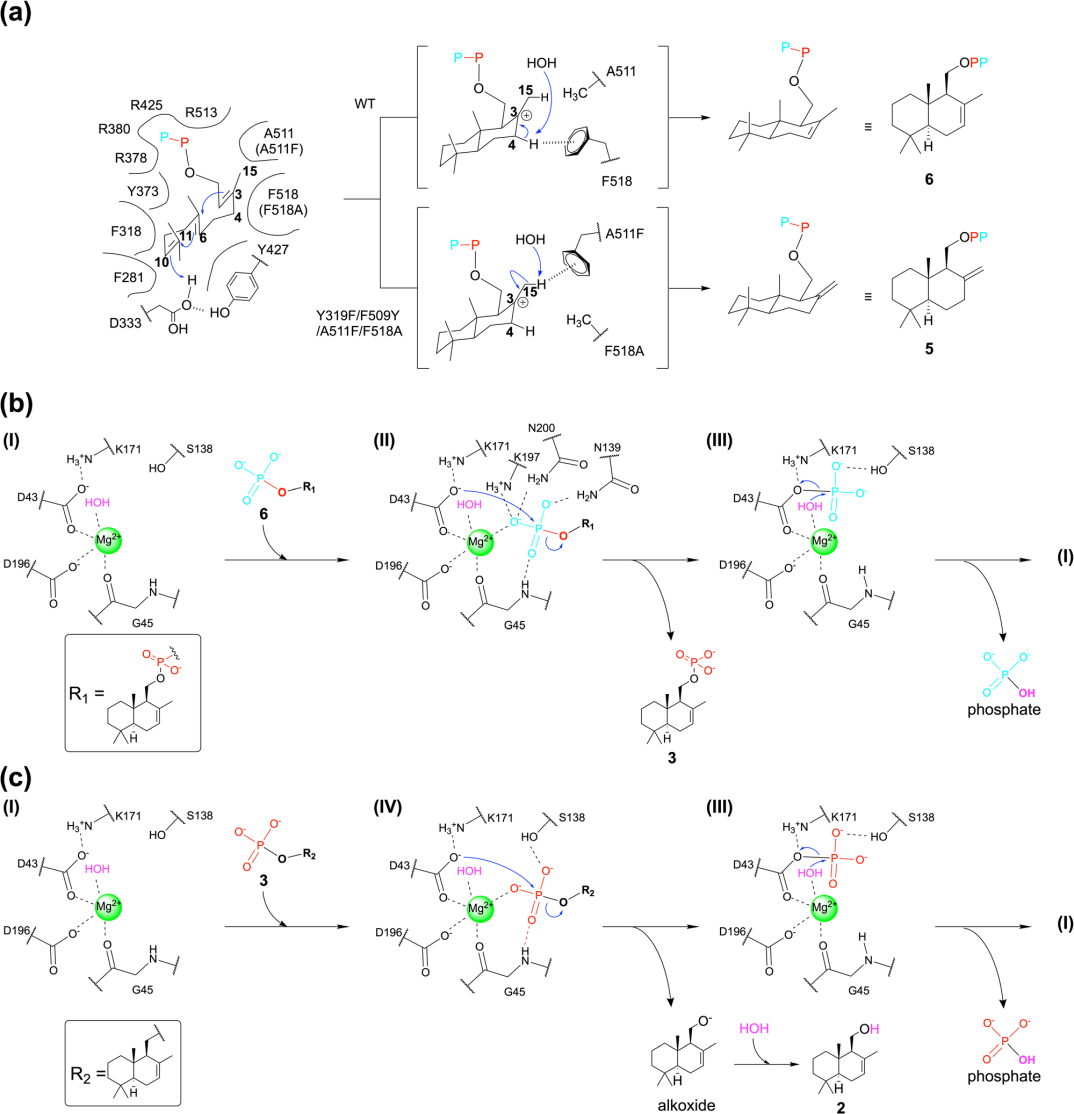
Reaction mechanism of AsDMS. (a) Predicted cyclization mechanism in the TCβ domain in wild-type and variant AsDMS. (b and c) Predicted first and second dephosphorylation mechanisms in the HAD domain of AsDMS. The blue arrows indicate the direction of electron transfer.

Based on these results, we propose a detailed catalytic mechanism for AsDMS. Initially, 1 binds within the TCβ domain, adopting an S-shaped conformation (Fig. 6a). Subsequently, residue D333, which is activated by residue Y427, catalyzes the protonation at the C10 position of 1, triggering a cascade reaction that leads to the formation of a bicyclic carbocation intermediate. The cyclization event might be finally quenched by a water molecule. Given the results of amino acid-switching experiments with the fungal 4-synthase and the resonance forms of the carbocation intermediate (Scheme S2), it is suggested that selective production of 6 or 5 is determined by C–H⋯π interactions with either F518 or A511F.^44^ It subsequently binds to the HAD catalytic domain by coordinating with Mg^2+^. Residue D43, which is activated by the interaction with residue K171, performs a nucleophilic attack on pyrophosphate substrate 6, releasing monophosphate product 3 and phosphate (Fig. 6b, state I–III). Subsequently, D43 nucleophilically attacks on the substrate 3, releasing alkoxide and phosphate. The alkoxide intermediate is immediately attacked by a water molecule, resulting in the production of 2 (Fig. 6c, state I–III). Consistent with general HAD enzyme catalytic mechanisms,^54,55^ it has been proposed that D43 is regenerated by a nucleophilic attack from a Mg^2+^-coordinated water molecule,^47,54,56^ enabling enzymatic turnover (Fig. 6b, state III and Fig. 6c, state III). Based on experiments using ^18^O-labeled water, Osika *et al.* explained that the hydroxyl group of 2 originates from 1, supporting the mechanism proposed in this study.^57^

Crystal structures of AsDMS bound to substrates 1 and 3 revealed distinct catalytic pockets within the TCβ and HAD domains. At first glance, this observation seems paradoxical when considering the higher catalytic efficiency of AsDMS relative to that of the single-catalytic-site enzyme *Valeriana officinalis* DMS,^31^ which directly produces 2 (Fig. S20a and b). However, given the dimeric structure of AsDMS, the spatial arrangement of the TCβ and HAD domains may facilitate substrate channeling. In the monomeric state, the domain pockets are oriented back-to-back. However, upon dimerization, the orientations of the pockets are improved (Fig. S20c and d). This spatial configuration enables efficient substrate transfer and promotes inter-subunit cooperation. Supporting this model, electrostatic surface analysis of the AsDMS dimer revealed a positively charged interface between the TCβ and HAD domains. This electrostatic environment may guide the negatively charged intermediate 6 between the domains, reducing the diffusion distance and minimizing its escape into the bulk solution. Together, these molecular features may contribute to the catalytic efficiency of AsDMS in producing 2. Notably, similar substrate-channeling mechanisms have been reported for other enzyme systems.^58–61^ This attractive system has the potential to enhance the catalytic efficiency of the enzyme and will be a subject to be addressed in future studies.

## Conclusions

In this study, we performed co-crystal structure analyses and biochemical characterization of site-specific variants to elucidate the structure–function relationships of AsDMS. Our results demonstrate that AsDMS is a bifunctional dimeric enzyme, comprising a protonation-initiated sesquiterpene cyclase (TCβ) domain and a metal-dependent HAD domain responsible for the stepwise release of phosphate to produce compound 2. AsDMS is the first HAD-TCβ enzyme for which the binding conformations and interactions of its physiological substrates (1 and 3) have been elucidated at the atomic level. Additionally, we engineered AsDMS from 2-synthase into 4-synthase by introducing four characteristic active-site residues, guided by structural modeling of fungal HAD-TCβ enzymes using AlphaFold2. The proposed molecular mechanism is likely conserved among bacterial HAD-TCβ enzymes. Furthermore, our study provides insights into the molecular evolution of bacterial and fungal HAD-TCβs, suggesting differences in linker structures, domain orientations, and oligomeric states of fungal HAD-TCβ enzymes. However, several questions remain to be addressed, such as the precise molecular mechanisms underlying fungal HAD-TCβ enzymes and the substrate-channeling mechanism within AsDMS. Addressing these challenges will facilitate the rational engineering of HAD-TCβ enzymes for the efficient biosynthesis of valuable sesquiterpenes.

## Author contributions

S. T. directed the study. K. F. performed enzyme purification, crystallography, and protein data analysis. K. F. and H. T. prepared chemicals. K. F., H. T., N. M., and N. N. Q. V. prepared the variants and performed enzyme assays. T. N. analyzed the structures of the small molecule. All the authors wrote the manuscript and discussed the data. K. F. and H. T. contributed equally to this study.

## Conflicts of interest

The authors declare no conflict of interest.

## Supplementary Material

SC-OLF-D5SC04719F-s001

SC-OLF-D5SC04719F-s002

SC-OLF-D5SC04719F-s003

SC-OLF-D5SC04719F-s004

## Data Availability

The coordinate and structure factor data for the AsDMS d18/D333N variant, 1-bound form, and 3-bound form were deposited in the Protein Data Bank under the accession codes 9M7D, 9M7F, and 9M7E, respectively. SI, including methods, supplementary figures, and tables, is available. See DOI: https://doi.org/10.1039/d5sc04719f.
