# Supplementary material for: Structural insights into a bacterial terpene cyclase fused with haloacid Dehalogenase-like phosphatase
Source: Chem Sci. 2025 Jul 28;16(34):15310–9. doi: 10.1039/d5sc04719f (PMC12365925; doi:10.1039/d5sc04719f)
Supplement: SC-016-D5SC04719F-s004 [file SC-016-D5SC04719F-s004.pdf]

Supplementary Information (SI) for Chemical Science.
This journal is © The Royal Society of Chemistry 2025

Supporting information

Structural Insights into a Bacterial Terpene Cyclase Fused with Haloacid Dehalogenase-like Phosphatase

Keisuke Fujiyama, [+] Hiroshi Takagi, [+] Nhu Ngoc Quynh Vo, Naoko Morita, Toshihiko Nogawa, and Shunji Takahashi*

[*] Dr. K. Fujiyama, H. Takagi, Dr. N. N. Q. Vo, N. Morita, Dr. S. Takahashi

Natural Product Biosynthesis Research Unit, RIKEN Center for Sustainable Research Science

2-1 Hirosawa, Wako, Saitama, 351-0198, Japan

E-mail: shunjitaka@riken.jp

Dr. K. Fujiyama

Plant Chemical Genetics Research Team, RIKEN Center for Sustainable Research Science

1-7-22 Suehiro-cho, Tsurumi-ku, Yokohama, Kanagawa, 230-0045, Japan

Dr. T. Nogawa

Molecular Structure Characterization Unit, RIKEN Center for Sustainable Research Science

2-1 Hirosawa, Wako, Saitama, 351-0198, Japan

[†] These authors contributed equally to this work

TABLE OF CONTENTS

Supporting Methods

Vector construction 4

Preparation of variant AsDMS for crystallization 4

Purification of AsDMS and its variants 4-5

Crystallization of AsDMS d18/D333N variant and crystal sample preparation 5-6

Data collection and structural determination of AsDMS d18/D333N variant 6

Structural similarity analyses through bioinformatics 6-7

Sample preparation and gel filtration analysis of the other bacterial DMSs 7

Enzyme assays of AsDMS 7

Divalent metal dependency of AsDMS 8

GC/MS analysis 8

MESG assays 8

Isolation and structure elucidation of drimenyl monophosphate (3) 8-9

Conversion of 3 to drimenol (2) 9

Docking simulation of 6 in the AsDMS HAD domain 9-10

Supporting Tables

Table S1. Primers used in this study. 11-13

Table S2. Crystallographic statistics. 14

Table S3. 500 MHz ^{1}H- and 125 MHz ^{13}C-NMR chemical shifts of isolated **3** 15

Supporting Figures

Fig. S1 Disorder and secondary structure predictions of wild-type AsDMS. 16

Fig. S2 Gel filtration analysis for bacterial DMSs. 17

Fig. S3 Structural similarity analysis of the AsDMS HAD domain. 18

Fig. S4 Structural similarity analysis of the AsDMS TCβ domain. 19

Fig. S5 Comparison of AsDMS HAD domain with the HAD domains of fungal HAD-TCβs and related HAD enzymes. 20

Fig. S6 Substrate recognition by class II sesquiterpene cyclases. 21

Fig. S7 Time-course activity measurements of AsDMS TCβ domain variants. 22

Fig. S8 Alignment with other bacterial DMSs. 23

Fig. S9 Alignment with other fungal DMSs. 24

Fig. S10 Pocket surface views of the TCβ domain in the **1**-bound structure. 25

Fig. S11 NMR spectra of **3** in D_2O. 26-27

Fig. S12 High-resolution GC/MS analysis of purified **3**. 28

Fig. S13 Enzymatic conversion of **3** to **2**. 29

Fig. S14 Divalent metal dependency of AsDMS. 30

Fig. S15 The MESG assay system. 32

Fig. S16 Detection of AsDMS activity as the production of Pi by MESG assay. 33

Fig. S17 HR-ESI MS analysis of an AsDMS reaction intermediate. 33-34

Fig. S18 Kinetic analysis of the AsDMS HAD domain using **3**. 35

Fig. S19 Docking simulation of the compound **6** in the AsDMS HAD domain. 36

Fig. S20 Putative substrate channeling system of AsDMS. 37

Scheme S1 Scheme of the overall AsDMS reaction. 38

Scheme S2 Resonance forms of the cyclized intermediate in the TCβ domain. 39

Supporting References 40-41

Vector construction

The AsDMS vector, which was constructed in a previous study,[1] was used to generate all variants and express the enzymes. Mutations were introduced via inverse PCR using primers for site-directed mutagenesis (Table S1). After amplification, the PCR products were digested with the restriction enzyme DpnI, *Escherichia coli* DH5a was transformed using the PCR products for a general heat shock method, and vector cloning was performed. Only Y427F variant of AsDMS and its vector were obtained using a DNA synthesis service (GenScript).

Regarding the vectors for glutathione-*S*-transferase (GST)-tagged AsDMS homolog enzymes (As119DMS and FeDMS), the cut vector of pET41a(+) and the enzyme DNAs for an insert were amplified using PCR with the primers (Table S1), and then GST-fused enzyme vectors were prepared using SLiCE methods[2,3]. pET41a(+) was modified with a His-tag at the N-terminus of GST.

Preparation of variant AsDMS for crystallization

Based on predictions of the secondary structure and disordered region, AsDMS has a long-disordered region at the N-terminus. Additionally, 14 N-terminal residues were not conserved among the marine bacterial DMSs (**Fig. S18**). Hence, the N-terminus-truncated variants d12, d14, d16, d18, and d34 were constructed. Based on its expression in *E. coli* BL21(DE3), d18, which exhibited good enzyme expression, was used to introduce the D333N mutation. The primers used for mutagenesis are listed in Table S1. The resulting variant enzyme (AsDMS d18/D333N) was used for the crystallization experiments.

Purification of AsDMS and its variants

AsDMS and its variants were expressed in *E. coli* BL21(DE3) star strain using the pET28b(+) vector, similar to the expression system used in a previous study.[1] *E. coli* was transformed with a heat shock method, and transformants were cultured in Luria-Bertani medium with 50 μg/mL kanamycin at 37°C overnight. Cultured cells were inoculated into the terrific broth with 50 μg/mL kanamycin and cultured at 37°C until the optical density at 600 nm had achieved 0.5. Then, 0.5 mM isopropyl-β-D-thiogalactopyranoside was added, and cells were cultured for 24 h at 18°C. After culturing *E. coli*, the cells were collected through centrifugation at 9,000 × *g* for 5 min at 4°C and stocked at −80°C until use.

The collected cells were suspended in buffer A (50 mM Tris-HCl [pH 8.0], 500 mM NaCl, 20% [v/v] glycerol) with 1 mg/mL lysozyme (Sigma-Aldrich), and a small amount of powdered DNase (Worthington) and disrupted through sonication on ice. Then, the supernatant, which was obtained through centrifugation (13,000 × *g*, 30 min, 4°C), was loaded into a Ni-NTA agarose column (Qiagen), and enzymes were roughly purified using wash buffer (buffer A with 30 mM imidazole) and elution buffer (buffer A with 200 mM imidazole) at 4°C. For the activity assay, purification was completed at this step, and the enzyme samples were concentrated using a 30 kDa cut-off Amicon Ultra-15 concentrator (Merck). Concentrated samples were stocked at −80°C after flush-freezing using liquid nitrogen.

For the crystallization, purified enzymes were digested by thrombin (nacalai tesque) with dialysis using buffer A at 4°C for 12 h, then undigested enzyme and his-tag were removed using a fresh Ni-NTA-agarose column. Finally, the enzyme was purified using HiLoad Superdex200 26/600 (Cytiva) gel filtration with buffer A (50 mM Tris-HCl [pH 7.5], 200 mM NaCl, and 12.5 mM [0.5 CM] *n*-octyl-β-D-glucoside. Crystallization samples were concentrated to 10 mg/mL using a 30 kDa cut-off Amicon Ultra-15 concentrator (Merck) and stocked at 4°C or −80°C after frozen using liquid nitrogen until use.

Crystallization of AsDMS d18/D333N variant and crystal sample preparation

All crystallization experiments were performed by mixing 2 μL of the 10 mg/mL ligand-free AsDMS d18/D333N variant protein solution with 2 μL of each reservoir solution with the sitting drop vapor diffusion method at 20°C. The protein crystals of the high-resolution data for the determination of the initial phase were obtained by mixing with a reservoir solution containing 95 mM MES-NaOH (pH 6.5), 5 mM MES-NaOH (pH 7.0), and 1.72 M ammonium sulfate and growing within two weeks. The obtained crystals were soaked into a cryoprotectant solution containing 50 mM MES-NaOH (pH 6.5), 500 mM NaCl, 2.4 M ammonium sulfate, and 16% (v/v) glycerol; then the crystals were flash-cooled by plunging into liquid nitrogen and preserved in liquid nitrogen. Crystals of **1**- and **3**/Ca^{2+}-bound compounds were prepared using soaking methods. For **1**-bound crystals, obtained apo-formed protein crystals were soaked into 100 mM MES-NaOH (pH 6.5), 500 mM NaCl, 2 M ammonium sulfate, 12% (v/v) glycerol, 5% (v/v) methanol, and 7.1 mM **1** for 1 h at room temperature. For **3**/Ca^{2+}-bound crystals, apo-

formed crystals were firstly soaked into 3 μL of a Ca^{2+} solution containing 100 mM MES-NaOH (pH 6.5), 500 mM NaCl, 2.8 M ammonium sulfate, and 1 mM $CaCl_2$ for 30 min at room temperature, then 3 μL of **3** solution containing 500 mM NaCl, 2.27 M ammonium sulfate, and 10 mM **3** was added to the Ca^{2+} solution with crystals, and it was left to stand for 30 min at room temperature. The prepared **1**- and **3**-bound crystals were flash-cooled using the same method as that used for the apo-formed crystals.

Data collection and structural determination of AsDMS d18/D333N variant

All X-ray diffraction experiments were performed at 100 K at the Photon Factory BL1A and BL17A (Ibaraki, Japan). The datasets were integrated and scaled using the XDS package[4] and AIMLESS[5] from the CCP4 suite. The initial phase was determined using the molecular replacement method in PhaserMR[6] from the CCP4 suite with the AsDMS monomer structure predicted by AlphaFold2[7] as the structural template model. The results showed that there were two molecular models in the asymmetric unit cell and that the models had a slight misalignment of the residues in the HAD domain. Therefore, further model building was manually performed with COOT (ver.0.9.8.7),[8] and model refinement was performed using Refmac5[9] and Phenix. Refine.[10] The obtained initial model was used for the phase determination of the high-resolution structure of AsDMS with molecular replacement. Additionally, in **1**- and **3**-bound models of AsDMS, phase determination, model building, and refinement were performed in the same manner as in the high-resolution structure. Ramachandran plot analysis showed that the residues of AsDMS d18/D333N, **1**-bound form, and **3**-bound form in the favored (allowed) region were 97.35 (2.55), 96.28 (3.53), and 96.25 (3.75) %, respectively. The data collection and final refinement statistics are summarized in Table S2. The model quality was validated using MolProbity[11] in the PHENIX package. Polder maps[12] of **1** and **3** were calculated using PHENIX. All structural model pictures used in this study were prepared using PyMOL.[13]

Structural similarity analyses through bioinformatics

For this analysis, we used the split models of HAD (residue: 21 to 225) and TCβ (residue: 235 to 536) domains in chain B, which had no disorder region in the model. Structural searches were performed using PDBeFold in service of the Protein Data Bank in Europe.[14,15] As analysis conditions, the lower acceptable matches of the query and

target proteins were 50% and 70%, respectively. The searched targets were selected based on protein data released in the PDB by December 1st, 2022. The selection of results was defined as follows: matched models were sorted in descending order of the RMDS score, and the highest-scoring model was selected only when there were some PDB IDs in a protein.

Sample preparation and gel filtration analysis of the other bacterial DMSs

GST-fused AsDMS homolog enzymes were prepared using the same expression and purification methods used for the AsDMS variants. Then, thrombin digestion of GST-fused enzymes with dialysis at 4°C overnight. Next, all enzymes (AsDMS, AsDMS d18/D333N variant, As119DMS, and FeDMS) were loaded onto HiLoad Superdex200 26/600 and analyzed using gel filtration buffer B (50 mM Tris-HCl [pH 7.5], 200 mM NaCl, and 20% [v/v] glycerol). Bovine serum albumin (BSA), which was used as a 66 kDa standard protein, was purchased (nacalai) and used. The dimerization of BSA is well known; therefore, the BSA dimer was used as a 132 kDa standard protein[16].

Enzyme assays of AsDMS

The activity of the TCβ domain of AsDMS was measured as previously described with minor modifications[1]. Briefly, the reaction was initiated by adding purified proteins (final 400 nM) to a reaction mixture (200 μL) of 50 mM Tris-HCl (pH 8.0), 2 mM $MgCl_2$, 1 mM dithiothreitol (DTT), and 100 μM farnesyl pyrophosphate (**1**). The reaction mixture was incubated at 30°C for 0–60 min. The reactions were terminated by adding 20 μL of 1 M NaOH. The aqueous layer was then extracted twice with 200 μL of hexane/ethyl acetate (1:1, v/v). The combined extract was analyzed using GC/MS.

The HAD domain activity of AsDMS was measured under the following conditions: The reaction mixture (200 μL) contained 50 mM Tris-HCl (pH 7.5), 10 μM $MgCl_2$, 1 μM DTT, and 1-100 μM **3**. After incubation at 30°C for 5 min, 20 nM enzyme (wild-type AsDMS or the HAD domain variants) was added to the reaction. The reaction product was extracted twice with 200 μL of ethyl acetate. The organic layer was combined and dried over anhydrous $MgSO_4$, and the samples were analyzed using GC/MS.

Divalent metal dependency of AsDMS

To evaluate divalent metal-dependency, AsDMS was incubated in a reaction mixture (200 µL) containing 50 mM Tris-HCl (pH 8.0), 2 mM divalent metal ions ($MgCl_2$, $MnCl_2$, $NiCl_2$, $CoCl_2$, or $CaCl_2$), 1 mM DTT, 100 µM **1**, and 200 nM purified enzyme. The reaction was conducted at 30°C for 1 h. A negative control reaction was performed in the presence of 1 mM ethylenediaminetetraacetic acid (EDTA) or in the absence of divalent metal ions. Extraction of the reaction products and GC/MS analysis were conducted using the workflow described above.

GC/MS analysis

The reaction products were analyzed using GC/MS as previously described.[1] The amount of reaction product was measured as the peak area using MSD ChemStation G1701EA E.02.02.1431 GC/MS software (Agilent). A standard curve for (–)-drimenol was obtained to calibrate the raw data.

MESG assays

The HAD domain phosphatase activity in wild-type AsDMS, AsDMS D333N variant, and D43A variant was evaluated using 7-methyl-6-thioguanosine (MESG, **7**).[17] Inorganic pyrophosphatase and bacterial purine nucleoside phosphorylase were obtained from commercial sources. Enzyme stock solutions were prepared by dissolving enzymes in 50 mM Tris-HCl (pH 7.5], 500 mM NaCl, and 20% [v/v] glycerol. Stock solutions (1 mM **7**, 2 mM $MgCl_2$, 10 mM sodium pyrophosphate [PPi], 10 mM **1**, and 10 mM **3)** were dissolved in water. The reaction mixture (200 µL) is shown in **Fig. S15a**. The enzyme reaction was conducted at 30°C in a quart cell, and the absorbance at 360 nm was monitored every 5 s for 15 min. The absorbance was measured using a spectrophotometer (V-630BIO, JASCO).

Isolation and structure elucidation of 3

Wild-type AsDMS reaction was conducted at 30°C in the reaction mixture containing 50 mM Tris-HCl (pH 7.5), 500 mM NaCl, 20% (v/v) glycerol, 10 mM EDTA-2Na (adjusted pH 8.0), 1 mM **1,** and 100 µM enzyme. After incubation for 6 h, an equal volume of acetonitrile was added to denature the enzyme. Acetonitrile was removed using the GeneVac software. The solution was centrifuged at 13,000 × *g* for 15 min at 4°C; the

supernatant was collected. 100 mM $CaCl_2$ was added, and the mixture was cooled on ice for 30 min. The suspension was centrifuged at 13,000 × *g* for 30 minutes at 4°C, and the slag was washed with distilled water. The slag was collected through centrifugation at 13,000 × *g* for 30 min at 4°C. The collected slag was dissolved in 100 mM EDTA-2Na (pH 8.0). The supernatant obtained through centrifugation at 13,000 × *g* for 10 min at 4°C was applied on Supelco TLC cellulose (20 × 20 cm, Merck), and phosphorylated metabolite was separated using a solvent system (isopropanol:ethyl acetate:10 mM NH_4OH = 2:2:1). The enzyme reaction product was visualized using phosphomolybdic acid, and the region corresponding to the stained spot (Rf = 0.75) was eluted using a solvent (MeOH:10 mM NH_4OH = 7:3). The eluate was evaporated to remove the organic solvent, and the water residue was dried through lyophilization.

The number of phosphate groups in **3** was inferred to be one using ^{31}P-NMR measurements, and the structure of **3** was determined by analyzing ^{1}H-, ^{13}C-, HSQC NMR spectra, as well as high-resolution MS spectra, and by comparing its chemical shifts with those previously reported for **6**.[18] NMR and HR-MS spectra were measured using a JNM-ECA500 (JEOL) and Synapt G2 mass spectrometer (Waters), respectively.

Conversion of 3 to 2

The conversion of **3** to **2** by bacterial alkaline phosphatase (TaKaRa) and AsDMS D333N were conducted in a reaction mixture containing alkaline phosphatase buffer and 1 μM **3** according to the manufacturer's protocol. After the reaction at 37°C for 1 h, the reaction mixture (100 μL) was extracted twice with 100 μL of ethyl acetate. The organic layer was combined and dried with anhydrous $MgSO_4$, and the sample was analyzed using a 7890A GC system equipped with a 5975C inert XL EI/CI MSD with a Triple-Axis detector (Agilent technology).

Docking simulation of 6 in the AsDMS HAD domain

The structure model of **6**, which is the cyclized compound produced by the TCβ domain of AsDMS, was built and energy minimized using Chem3D v.23.1 (Revvity). The A-chain in the asymmetric unit of a crystal structure of **3**-bound AsDMS was utilized as a receptor template since it had no chain-braking. Additions of hydrogen atoms and charge of the divalent metal were performed using AutoDockTools (v.1.5.6).[19] The side chain of K197 was treated as flexible entities to facilitate the calculation. The docking

simulations were performed using AutoDock Vina[20] under the following conditions: calculation grid boxes was 46×56×48 $Å^3$, and the exhaustiveness value was 200.

Table S1 Primers used in this study.

Primers	Sequence
AsDMS-d12-fwd	GCAGCCATAACAACATGAAGGAAGTGCTGACCCCG
AsDMS-d12-rev	ATGTTGTTATGGCTGCCGCGCGGC
AsDMS-d14-fwd	GCAGCCATATGAAGGAAGTGCTGACCCCGGAAC
AsDMS-d14-rev	TCCTTCATATGGCTGCCGCGCGGC
AsDMS-d16-fwd	GCAGCCATGAAGTGCTGACCCCGGAACTGATCAC
AsDMS-d16-rev	AGCACTTCATGGCTGCCGCGCGGC
AsDMS-d18-fwd	GCAGCCATCTGACCCCGGAACTGATCACCAGTCTG
AsDMS-d18-rev	GGGGTCAGATGGCTGCCGCGCGGC
AsDMS-d34-fwd	GCAGCCATGATCGCTACGACACGATCGTTTTTGATCTGG
AsDMS-d34-rev	TAGCGATCATGGCTGCCGCGCGGC
AsDMS-D43A-fwd	CGTTTTTGCGCTGGGCGACGTTCTGCTCC
AsDMS-D43A -rev	TCGCCCAGCGCAAAAACGATCGTGTCGTAGCGATC
AsDMS-L44A-fwd	TTTTTGATGCGGGCGACGTTCTGCTCCAC
AsDMS-L44A-rev	CGTCGCCCGCATCAAAAACGATCGTGTCGTAGCG
AsDMS-G45A-fwd	TGATCTGGCGGACGTTCTGCTCCACTGGGAC
AsDMS-G45A-rev	AGAACGTCCGCCAGATCAAAAACGATCGTGTCGTAG
AsDMS-L49A-fwd	ACGTTCTGGCCCACTGGGACAGTGTGCATTTTAC
AsDMS-L49A-rev	CCCAGTGGGCCAGAACGTCGCCCAGATCAAAAAC
AsDMS-W51A-fwd	TGCTCCACGCGGACAGTGTGCATTTTACCAGTGAG
AsDMS-W51A-rev	CACTGTCCGCGTGGAGCAGAACGTCGCCC
AsDMS-V70A-fwd	CAAAATGGCGAAACACCCGGTGTGGCAAG
AsDMS-V70A-rev	GGTGTTTCGCCATTTTGCGAACGTCGTCGATG
AsDMS-W75A-fwd	ACCCGGTGGCGCAAGACCTCGAAAAGGGCC
AsDMS-W75A-rev	GGTCTTGCGCCACCGGGTGTTTCACCATTTTGC
AsDMS-E79A-fwd	AGACCTCGCGAAGGGCCTCATCAATCAAGAATTTGC
AsDMS-E79A-rev	AGGCCCTTCGCGAGGTCTTGCCACACCGG
AsDMS-L107A-fwd	AGGAGATGGCGGAACTCAGCATCGCCAGC
AsDMS-L107A-rev	TGAGTTCCGCCATCTCCTTCAGCTTGCTGCAC
AsDMS-I111A-fwd	AACTCAGCGCGGCCAGCCTCCAAGTTAATCCG
AsDMS-I111A-rev	AGGCTGGCCGCGCTGAGTTCCAGCATCTCCTTC
AsDMS-S138A-fwd	ACTGTCTGGCGAACGTGGATCTCGAAAGCTTCAG
AsDMS-S138A-rev	TCCACGTTCGCCAGACAGTAGATCTGCTTGTCCTTTTTG
AsDMS-N139A-fwd	GTCTGAGCGCGGTGGATCTCGAAAGCTTCAGCTATC
AsDMS-N139A-rev	AGATCCACCGCGCTCAGACAGTAGATCTGCTTGTCC
AsDMS-V140A-fwd	GAGCAACGCGGATCTCGAAAGCTTCAGCTATCTG
AsDMS-V140A-rev	CGAGATCCGCGTTGCTCAGACAGTAGATCTGC
AsDMS-L148A-fwd	TCAGCTATGCGTATAAGCAGTTCGATTTTTGGAAATATTTCGAC
AsDMS-L148A-rev	GCTTATACGCATAGCTGAAGCTTTCGAGATCCACG
AsDMS-K171A-fwd	AGCTCCGCGCACCAAACCCGGACATCTTCCAGTATCTGATCAGC
AsDMS-K171A-rev	GGTTTGGTGCGCGGAGCTGGAGCAGCGCACTCACGTAAATG
AsDMS-D195A-fwd	CTTCATCGCCGACAAGAGCGAGAATCTGCAAG
AsDMS-D195A-rev	TCTTGTCGGCGATGAAGATGGTGCTCTTCGTG
AsDMS-D196A-fwd	CATCGACGCCAAGAGCGAGAATCTGCAAGAGG

AsDMS-D196A-rev	CGCTCTTGGCGTCGATGAAGATGGTGCTCTTC
AsDMS-K197A-fwd	TCGACGACGCAAGCGAGAATCTGCAAGAGGCG
AsDMS-K197A-rev	TTCTCGCTTGCGTCGTCGATGAAGATGGTGCTCTTC
AsDMS-F281A-fwd	GCAAAGAAATCGCCAGCACCGCCGTGATTCTGCATAGTTAT
AsDMS-F281A-rev	ATAACTATGCAGAATCACGGCGGTGCTGGCGATTTCTTTGC
AsDMS-F318A-fwd	TCCGTTGGTGTGCCTACAAAAACGAGGCCCGCCCGGATAAT
AsDMS-F318A-rev	ATTATCCGGGCGGGCCTCGTTTTTGTAGGCACACCAACGGA
AsDMS-Y319A-fwd	GTTGGTGTTTCGCCAAAAACGAGGCCCGCCCGGATAATTTC
AsDMS-Y319A-rev	GAAATTATCCGGGCGGGCCTCGTTTTTGGCGAAACACCAAC
AsDMS-F328A-fwd	GCCCGGATAATGCCCCGGACGATCTGGACACCACGAGTATG
AsDMS-F328A-rev	CATACTCGTGGTGTCCAGATCGTCCGGGGCATTATCCGGGC
AsDMS-D331A-fwd	ATTTCCCGGACGCCCTGGACACCACGAGTATGGTGCTCAGC
AsDMS-D331A-rev	GCTGAGCACCATACTCGTGGTGTCCAGGGCGTCCGGGAAAT
AsDMS-D331N-fwd	ATTTCCCGGACAACCTGGACACCACGAGTATGGTGCTCAGC
AsDMS-D331N-rev	GCTGAGCACCATACTCGTGGTGTCCAGGTTGTCCGGGAAAT
AsDMS-D333N-fwd	ACGATCTGAACACCACGAGTATGGTGCTCAGC
AsDMS-D333N-rev	CGTGGTGTTCAGATCGTCCGGGAAATTATCCGGG
AsDMS-Y373A-fwd	TCATCCAAGTTGCTTTCGACGACAATCGCCCACGTATCGAT
AsDMS-Y373A-rev	ATCGATACGTGGGCGATTGTCGTCGAAAGCAACTTGGATGA
AsDMS-R378A-fwd	TCGACGACAATGCCCCACGTATCGATGCCATCGTTGCCATC
AsDMS-R378A-rev	GATGGCAACGATGGCATCGATACGTGGGGCATTGTCGTCGA
AsDMS-R380A-fwd	ACAATCGCCCAGCCATCGATGCCATCGTTGCCATCAATGTG
AsDMS-R380A-rev	CACATTGATGGCAACGATGGCATCGATGGCTGGGCGATTGT
AsDMS-R425A-fwd	TGAAGGGCACGGCCTATTATCCAGCGCCGGACGTTTTTCTG
AsDMS-R425A-rev	CAGAAAAACGTCCGGCGCTGGATAATAGGCCGTGCCCTTCA
AsDMS-Y426A-fwd	AGGGCACGCGCGCTTATCCAGCGCCGGACGTTTTTCTGTTC
AsDMS-Y426A-rev	GAACAGAAAAACGTCCGGCGCTGGATAAGCGCGCGTGCCCT
AsDMS-F509A-fwd	TTTATGGTCTGGCTATCGCCCCACGTAGCAACACGTACTTC
AsDMS-F509A-rev	GAAGTACGTGTTGCTACGTGGGGCGATAGCCAGACCATAAA
AsDMS-A511G-fwd	GTCTGTTTATCGGCCCACGTAGCAACACGTACTTCGGCAGC
AsDMS-A511G-rev	GCTGCCGAAGTACGTGTTGCTACGTGGGCCGATAAACAGAC
AsDMS-R513A-fwd	TTATCGCCCCAGCCAGCAACACGTACTTCGGCAGCCGTGAA
AsDMS-R513A-rev	TTCACGGCTGCCGAAGTACGTGTTGCTGGCTGGGGCGATAA
AsDMS-S514A-fwd	TCGCCCCACGTGCCAACACGTACTTCGGCAGCCGTGAACTG
AsDMS-S514A-rev	CAGTTCACGGCTGCCGAAGTACGTGTTGGCACGTGGGGCGA
AsDMS-F518A-fwd	GCAACACGTACGCCGGCAGCCGTGAACTGAGTACGGCCTTC
AsDMS-F518A-rev	GAAGGCCGTACTCAGTTCACGGCTGCCGGCGTACGTGTTGC
AsDMS-Y319F-fwd	GTGTTTCTTCAAAAACGAGGCCCGCCC
AsDMS-Y319F-rev	CGTTTTTGAAGAAACACCAACGGAGCTTATTCTG
AsDMS-F509Y-fwd	TGGTCTGTATATCGCCCCACGTAGCAACAC
AsDMS-F509Y-rev	GGGCGATATACAGACCATAAACCGGCCAAC
AsDMS-A511F-fwd	TGTTTATCTTTCCACGTAGCAACACGTAC
AsDMS-A511F-rev	CTACGTGGAAAGATAAACAGACCATAAACCGGC
AsDMS-Y509Y/A511-fwd	TGGTCTGTATATCTTTCCACGTAGCAACACGTAC
AsDMS-Y509Y/A511-rev	GAAAGATATACAGACCATAAACCGGCCAAC
pET41a-cut-fwd	TAATGAAGCTTGCGGCCGCACTC

pET41a-cut-rev	ATGACTACCGCGTGGCACCAGAG
FeDMS-amplification-fwd	TGCCACGCGGTAGTCATATGAAAGAACTGAGCACGCCGGAGG
FeDMS-amplification-rev	GGCCGCAAGCTTCATTAGTTGCGCTTGCTCATCAGATTGATGGC
As119DMS-amplification-fwd	TGCCACGCGGTAGTCATATGAAAGAAGTGCTCACCCCAGAGCTC
As119DMS-amplification-rev	GGCCGCAAGCTTCATTAGCTCAGCAGATTCAGCGCTTCGAG
pET28b-cut-fwd	GAGGGTTGCTCGCGCTAACTCGAGCACCACCACCACCACCACTGAG
pET28b-cut-rev	CGGACTAGCGTTCATATGGCTGCCGCGCGGCACCAG
SsDMS-amplification-fwd	ATGAACGCTAGTCCGACCCCTACAGC
SsDMS-amplification-rev	GCGCGAGCAACCCTCTGGATCC

Table S2 Crystallographic statistics.

	AsDMS d18/D333N	**1**-bound form	**3**/Ca^{2+}-bound form
PDB Code	9M7D	9M7F	9M7E
Data Collection			
Beam source	BL-17A (Photon Factory)	BL-1A (Photon Factory)	BL-1A (Photon Factory)
Wavelength (Å)	1.000000	1.040000	1.030000
Resolution range (Å)	47.18 - 2.3 (2.382 - 2.30)	48.27 - 2.6 (2.693 - 2.60)	48.37 - 2.9 (3.004 - 2.90)
Space group	$P\,4_32_12$	$P\,4_32_12$	$P\,4_32_12$
Unit cell parameters			
a, b, c (Å)	a = b = 97.032, c = 405.183	a = b = 96.546, c = 401.409	a = b = 97.437, c = 403.197
α, β, γ (°)	α = β = γ = 90	α = β = γ = 90	α = β = γ = 90
Total reflections	1149521 (118665)	804631 (82866)	585326 (60259)
Unique reflections	87319 (8539)	59769 (5855)	44369 (4328)
Multiplicity	13.2 (13.9)	13.5 (14.2)	13.2 (13.9)
Completeness (%)	99.97 (100.00)	99.95 (100.00)	99.93 (99.98)
Mean I / sigma(I)	15.12 (1.61)	13.84 (1.85)	11.77 (1.16)
Wilson B-factor	44.73	53.56	71.84
R_{merge}	0.1531 (1.622)	0.1711 (1.324)	0.2125 (1.998)
R_{pim}	0.0440 (0.4500)	0.0482 (0.3635)	0.0606 (0.5536)
$CC_{1/2}$	0.999 (0.627)	0.998 (0.72)	0.997 (0.551)
Refinement			
Number of atoms	87306 (8539)	59759 (5855)	44356 (4328)
Protein	0.1837 / 0.2263	0.1929 / 0.2356	0.2016 / 0.2427
**1**	8917	8770	8521
**3**	8412	8397	8353
Others[a]	-	96	48
Water	-	-	40
RMSD	148	83	63
Bond lengths (Å)	357	194	17
Bond angles (°)			
Average B-factor	0.013	0.008	0.002
Protein	1.21	0.95	0.42
**1**	51.26	55.26	67.25
**3**	50.59	54.87	66.58
Others[a]	-	61.27	77.82
Water	-	-	103.99
Number of atoms	85.7	102.36	129.05
Protein	52.82	49.42	48.86

[a] Others include ions, small molecules, and undefined atomic models.

[b] $R_{factor}=\Sigma_{hkl}|F_{obs}(hkl)-F_{calc}(hkl)|/\Sigma_{hkl}F_{obs}(hkl)$, where F_{obs} and F_{calc} are observed and calculated structure factor amplitudes, respectively. R_{free} was the R factor calculated using 5% of the selected reflections not included for refinement.

Table S3 500 MHz ^{1}H- and 125 MHz ^{13}C-NMR chemical shifts of isolated **3.**

**3** (D_2O)*				**6****			
Position	δ^{13}C	δ^{1}H		Position	δ^{13}C	δ^{1}H	
1	39.0	1.93	2H	1	39.0	1.97	2H
2	18.1	1.39	1H	2	18.2	1.39	1H
		1.34	1H			1.14	1H
3	41.5	1.29	1H	3	41.5	1.29	1H
		1.06	1H			1.14	1H
4	32.3	-		4	32.1	-	
5	49.7	1.05	1H	5	49.5	1.06	1H
6	23.2	1.93	1H	6	23.0	1.92	1H
		1.78	1H			1.54	1H
7	124.0	5.54	1H	7	124.0	5.48	1H
8	134.9	-		8	134.7	-	
9	52.8	1.86	1H	9	54.4	1.97	1H
10	35.6	-		10	35.4	-	
11	63.2	3.97	1H	11	64.3	4.12	1H
		3.86	1H			3.98	1H
12	21.1	1.70	3H	12	21.2	1.73	3H
13	32.8	0.82	3H	13	32.6	0.81	3H
14	21.0	0.79	3H	14	21.2	0.85	3H
15	14.1	0.77	3H	15	14.0	0.81	3H

δ^{31}P (202.4 MHz, D_2O): 1.24 ppm

* ^{1}H-^{13}C attachments were determined with HSQC, and the position was determined by comparing chemical shifts with drimenyl pyrophosphate (**6**).

** 400 MHz ^{1}H, 100 MHz ^{13}C, D_2O.[18]

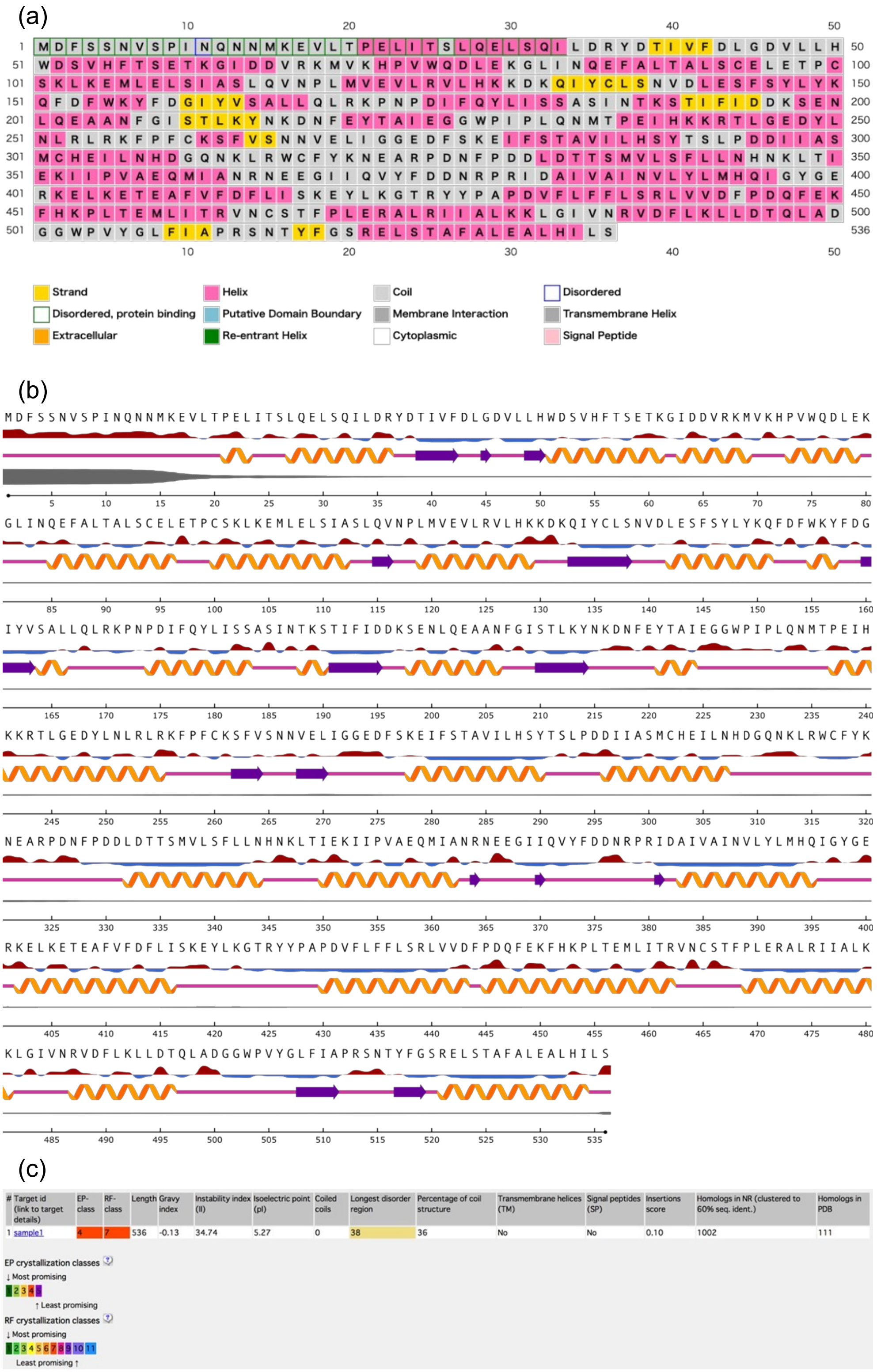

Fig. S1 Disorder and secondary structure predictions of wild-type AsDMS. (a) A result using PSIPRED 4.0[21] of wild-type AsDMS. (b) A result using NetSurfP 2.0[22] of AsDMS. (c) A result using XtalPred[23] of wild-type AsDMS.

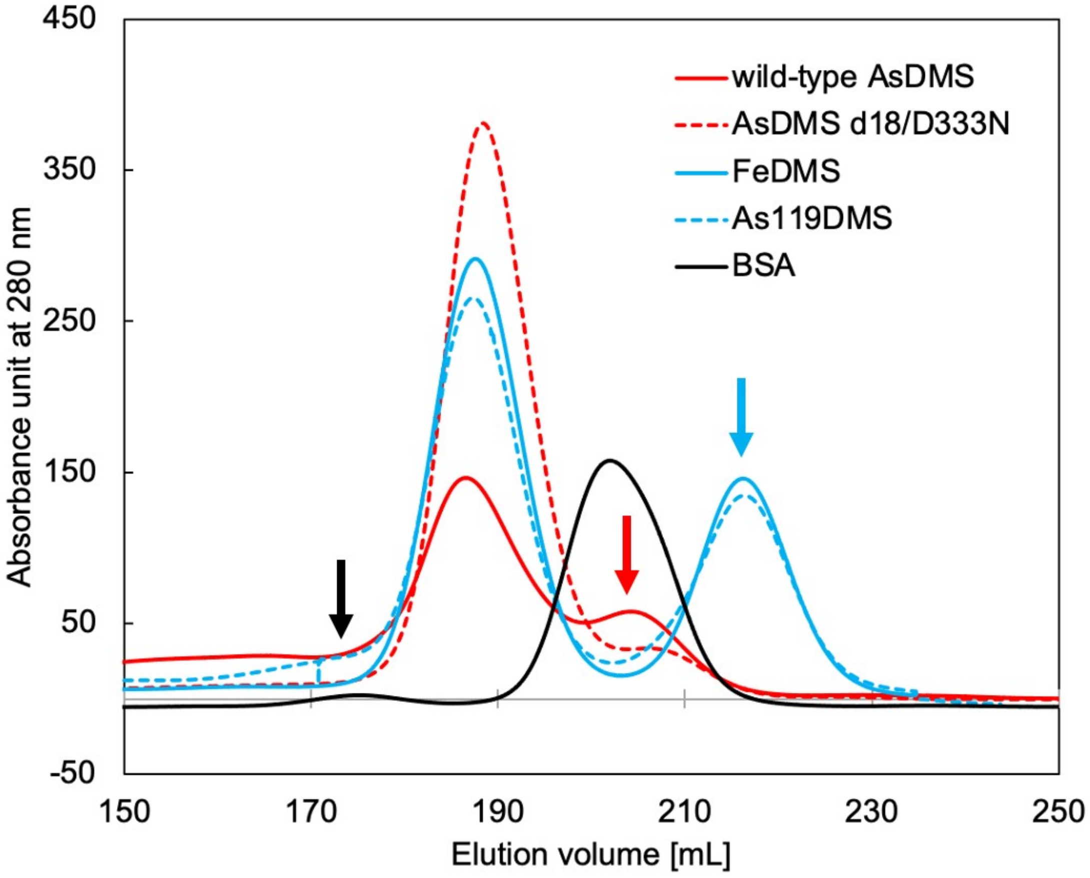

Fig. S2 Gel filtration analysis for bacterial DMSs. The black arrow indicates the BSA dimer. The cyan arrow indicates glutathione-*S*-transferase (GST), which was eliminated from FeDMS and As119DMS by thrombin digestion. The red arrow indicates the AsDMS monomer. The molecular weights of AsDMS, AsDMS d18/D333N, FeDMS, As119DMS, GST, and BSA are 62, 60, 60,60, 28, and 66 kDa, respectively

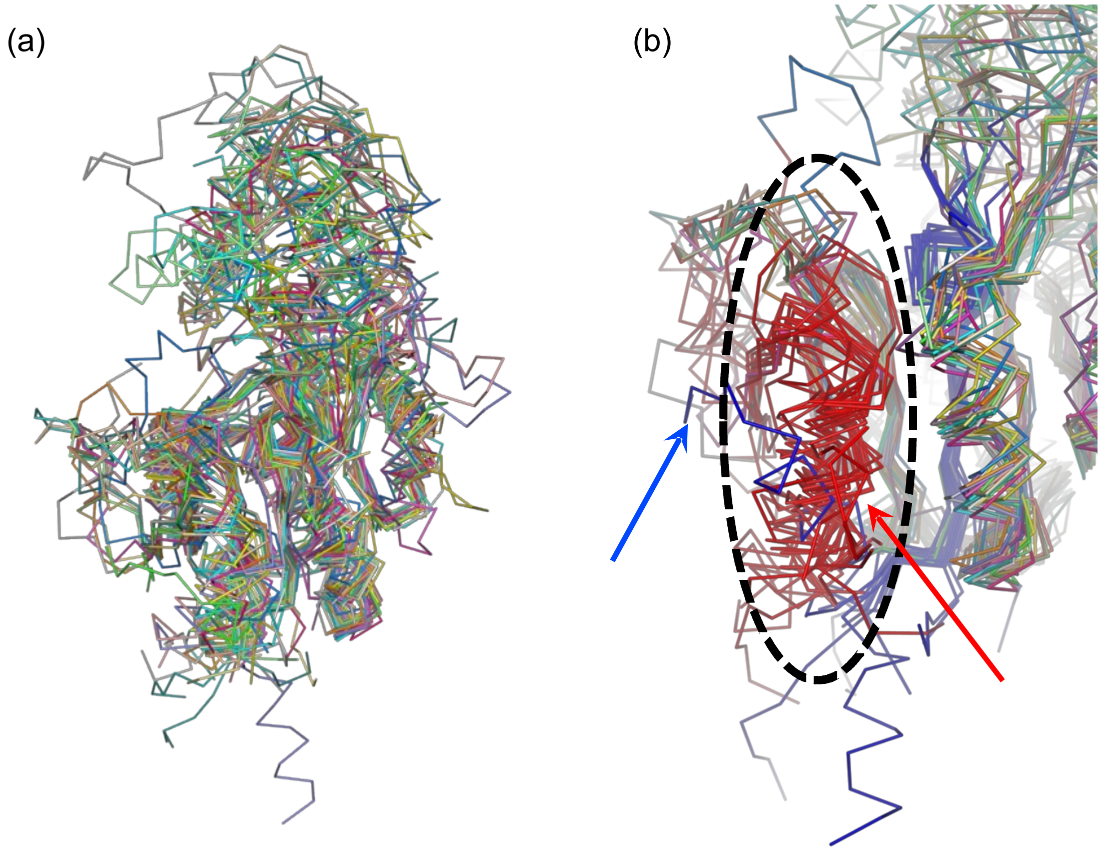

Fig. S3 Structural similarity analysis of the AsDMS HAD domain. (a) The structural alignment of 20 proteins at the top of the result for the HAD domain. The PDB IDs except for AsDMS were 5MWA, 4PNH, 3CNH, 1U7P, 2PR7, 2WM8, 3IB6, 3I76, 4YGQ, 2B0C, 2MU1, 5UJ0, 2PKE, 3UMC, 4DFD, 6Q7N, 2GFH, 3U26, 4KNW, and 6Q7P. An expanded view of the superposition. N- and C-termini of all HAD domains were colored blue and red, respectively. The blue arrow indicates the N-terminal helix of AsDMS HAD domain. The red arrow is the C-terminal helices of listed phosphatases. The dashed circle indicates a C-terminal helix region of general HAD enzymes.

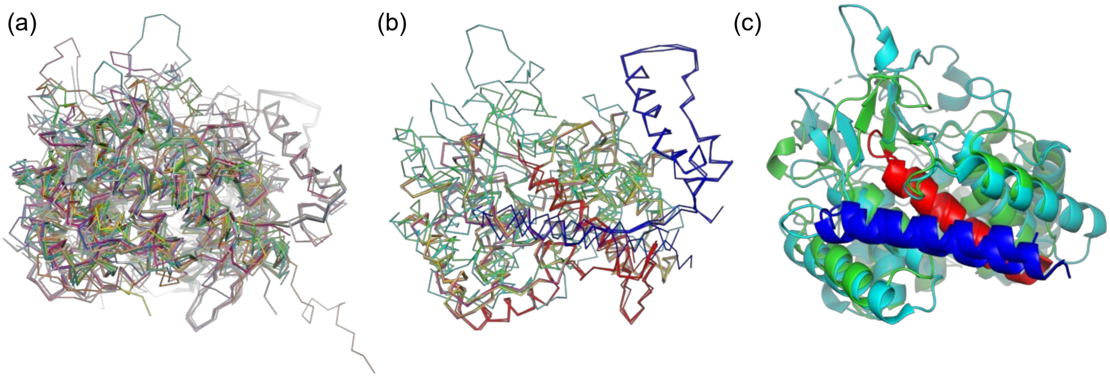

Fig. S4 Structural similarity analysis of the AsDMS TCβ domain. (a) The structural alignment of 20 proteins at the top of a result for the TCβ domain. The PDB IDs except for AsDMS were 6SBF, 6IJ1, 2PMV, 4CE7, 3REN, 5HOP, 3QWT, 1C3D, 3D5S, 2F0Y, 3RJ3, 1N95, 1FPP, 1QBQ, 2GOX, 1NL4, 3OED, 4M76, 1FT2, and 4ONT. (b) A superposition of terpenoid biosynthesis-related proteins to the TCβ domain of AsDMS. N- and C-termini were colored blue and red, respectively. The protein models used were PDB IDs 1FPP, 1QBQ, 1N95, 2F0Y, and 6SBF. (c) A superposition of merosterolic acid synthase (PBD ID: 6SBF)[24] and AsDMS TCβ domain, colored green and cyan, respectively. Their N- and C-termini colored blue and red, respectively.

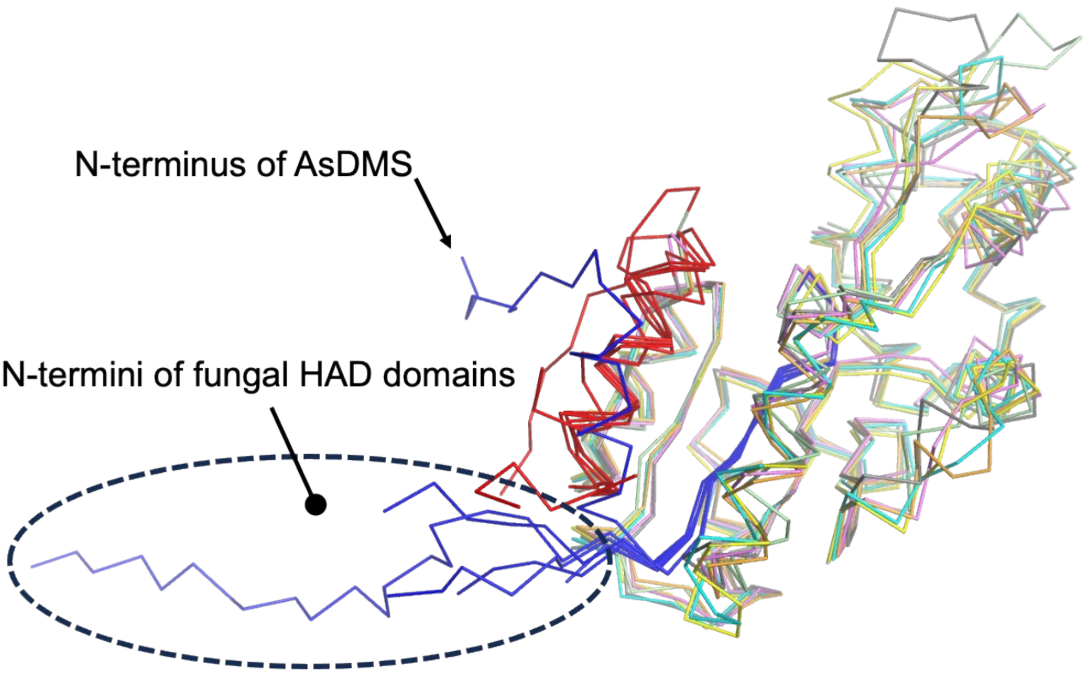

Fig. S5 Comparison of the AsDMS HAD domain with the HAD domains of fungal HAD-TCβs and related HAD enzymes. The structures of all fungal HAD-TCβs were generated using AlphaFold2[7]. The HAD domains derived from AsDMS (M1-E225, green), AncC[25] (M1-E213, orange), AstC[26] (M1-E198, yellow), DrtB[25] (M1-E199, cyan), AstI[26] (M1-I201, gray), and AstK[26] (M1-C196, pink), respectively. All N- and C-termini of HAD domains are colored blue and red, respectively.

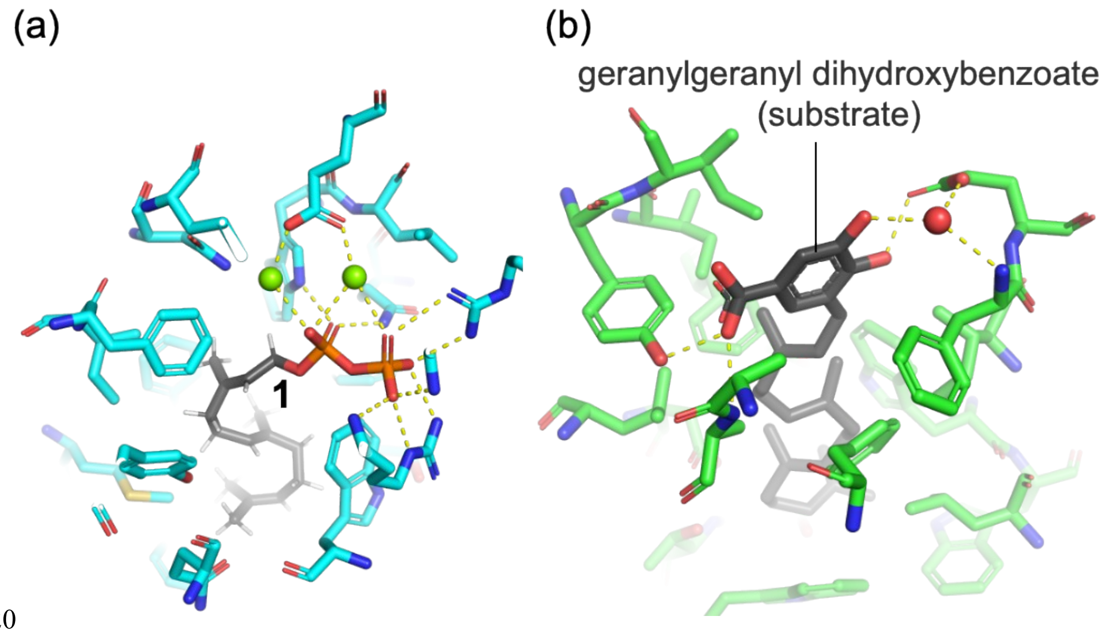

Fig. S6 Substrate recognition by class II sesquiterpene cyclases. (a) Recognition of the phosphate moiety in **1** by *Streptomyces showdoensis* DMS (PDB ID: 7XRA).[18] (b) Recognition of the hydrophilic moiety in a meroterpenoid substrate by merosterolic acid synthase (PDB ID: 6SBG).[24] The yellow dashed lines indicate hydrogen bonds. The red and green spheres represent water and Mg^{2+} molecules, respectively.

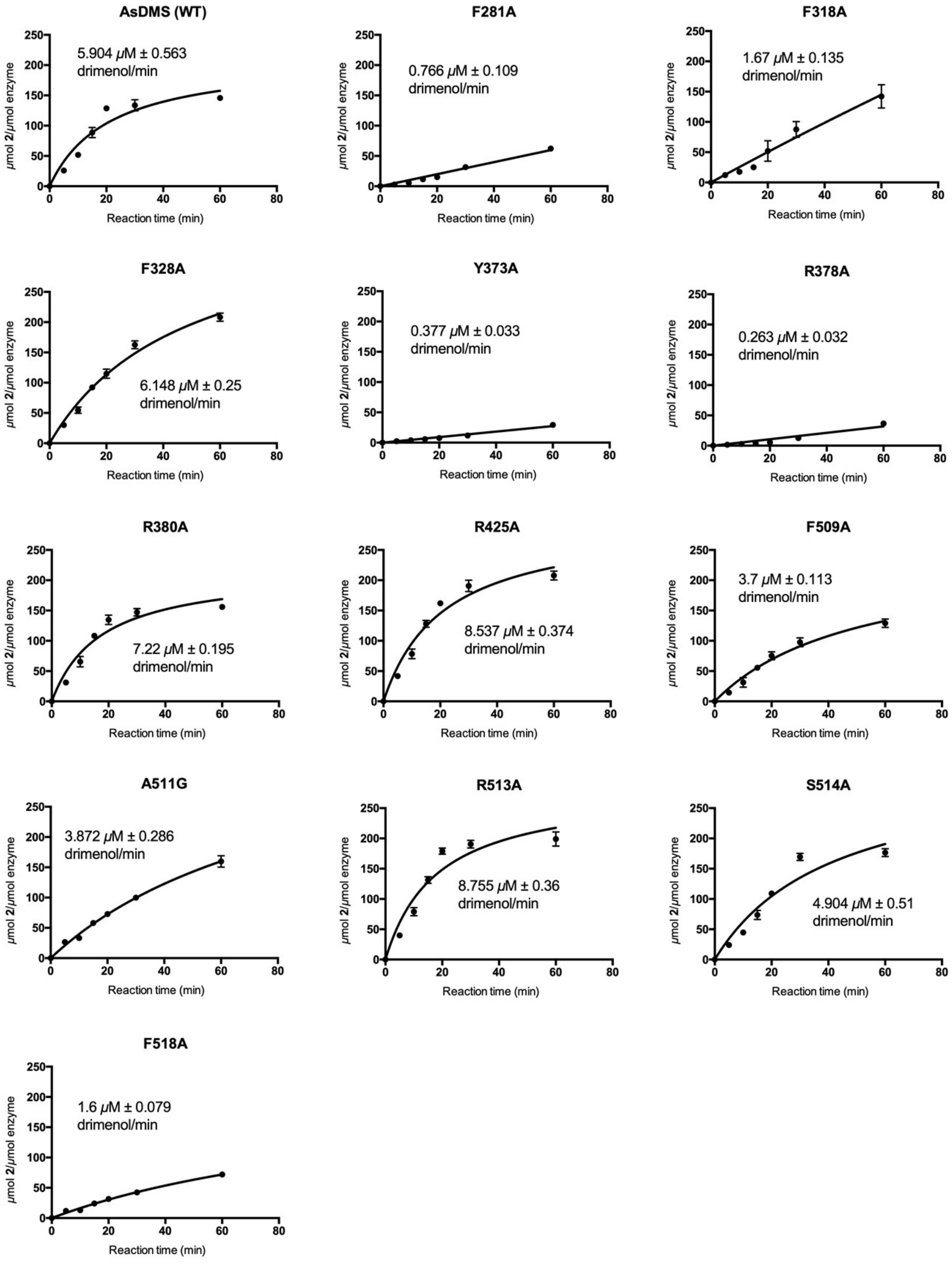

Fig. S7 Time-course activity measurements of wild-type AsDMS and TCβ domain variants. Data are given as means ± SD of three independent experiments performed with

the same preparation of each purified protein.

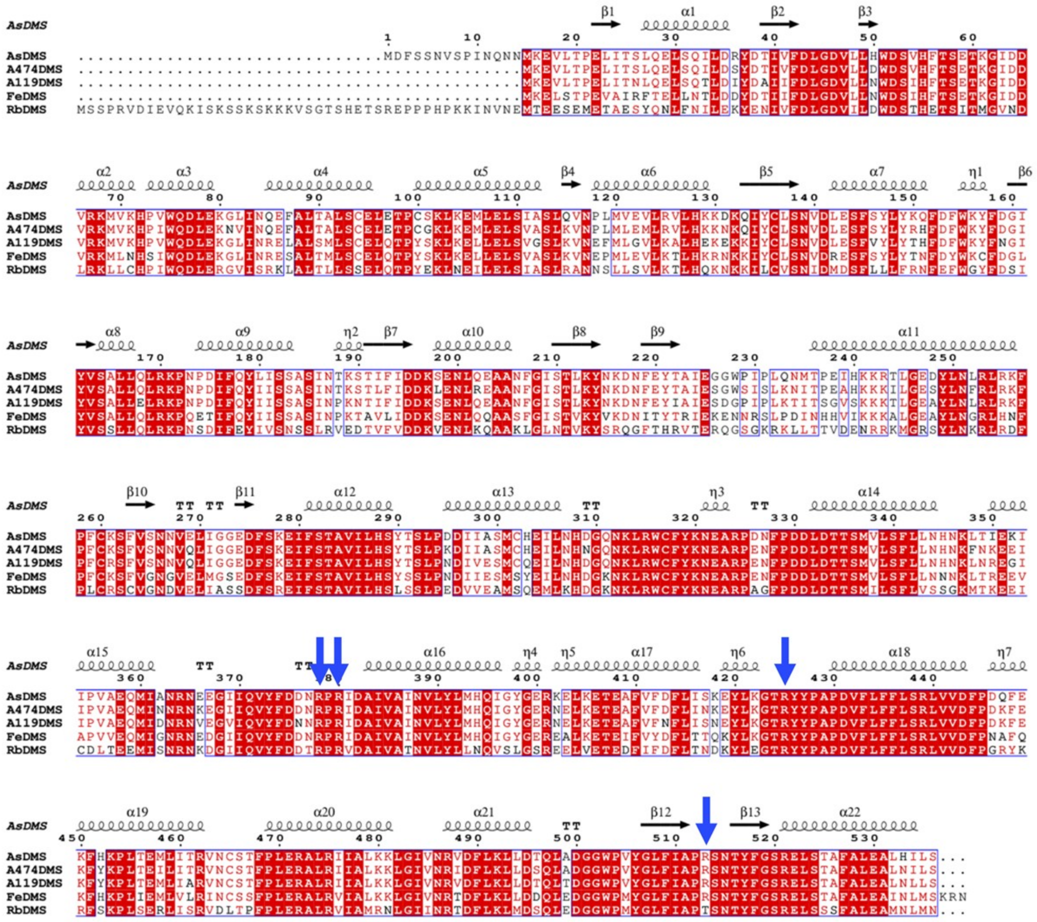

Fig. S8 Alignment with other bacterial DMSs. The alignment was generated by ESPript 3.0.[28] Blue arrows indicate the binding sites for phosphate moiety of **1** in TCβ domain of AsDMS.

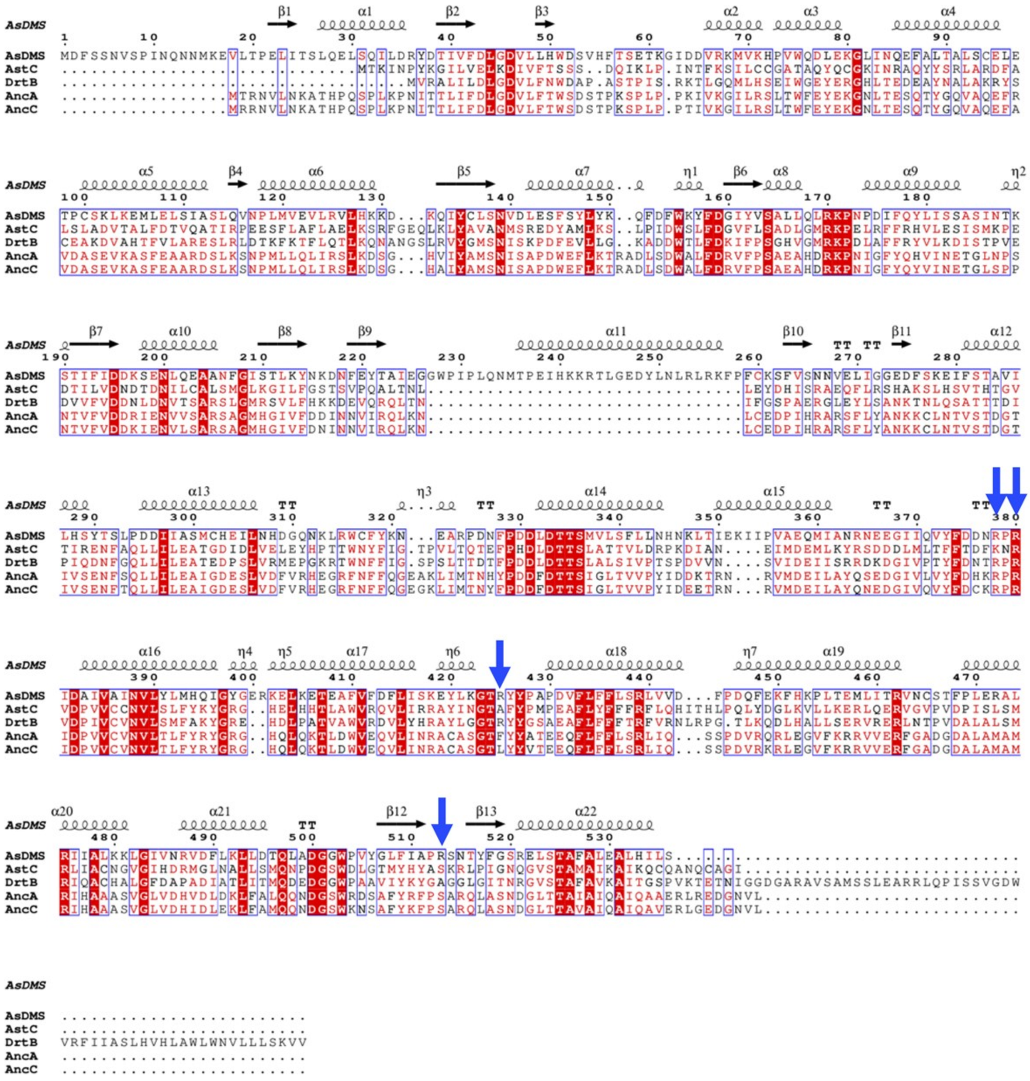

Fig. S9 Alignment with other fungal DMSs. The alignment was generated by ESPript 3.0.[28] Blue arrows indicate the binding sites for phosphate moiety of **1** in TCβ domain of AsDMS.

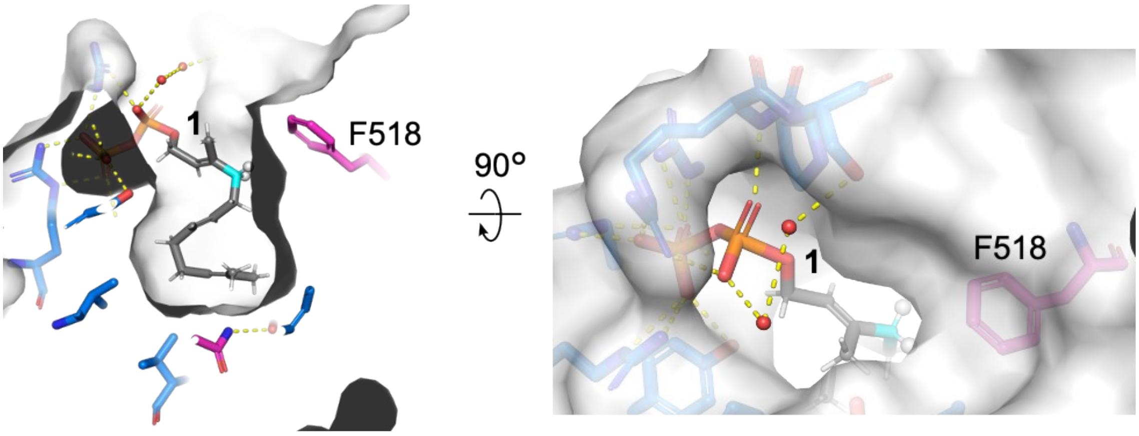

Fig. S10 Pocket surface views of the TCβ domain in the **1**-bound structure. The cyan-colored carbon in **1** indicates the C4 position of **1**, and the hydrogen atoms at C4 are represented as white spheres. F518 is colored magenta. The yellow dashed lines represent hydrogen bonds, and the red spheres represent ordered water molecules.

(a)

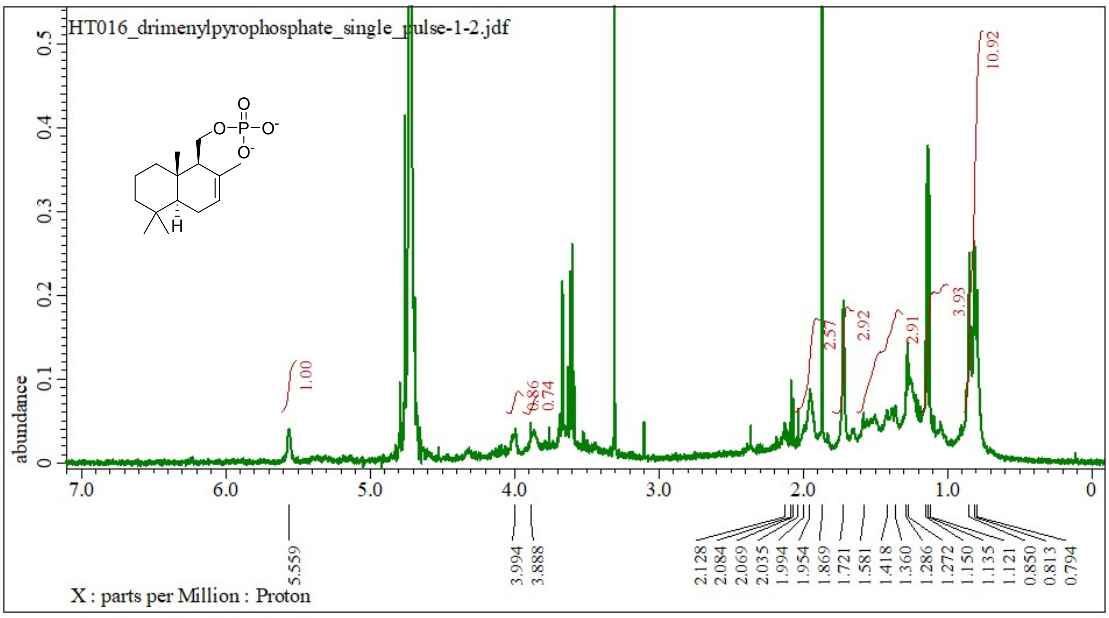

(b)

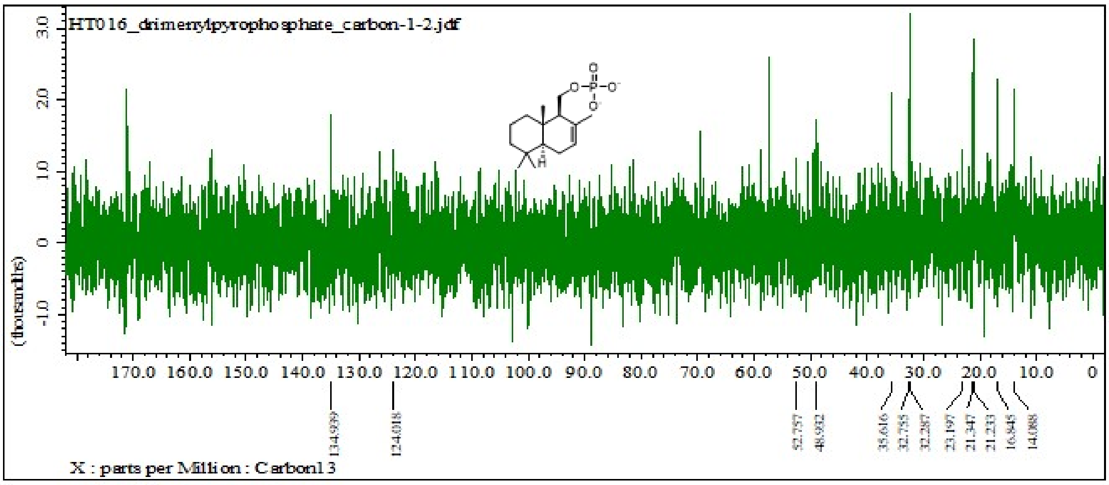

Continue on next page

(c)

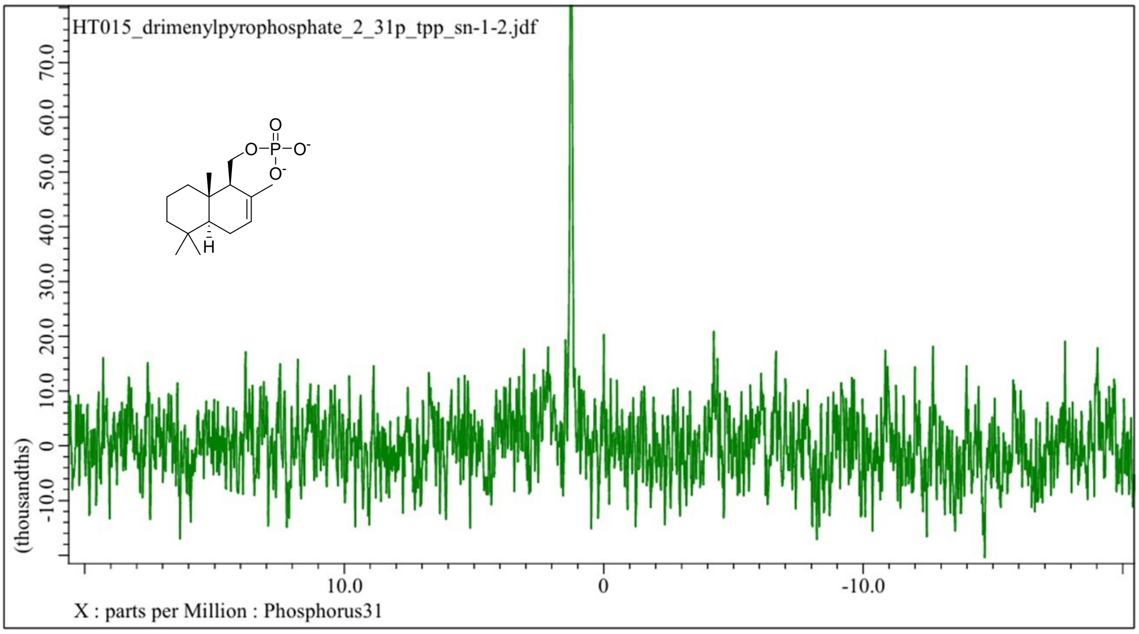

(d)

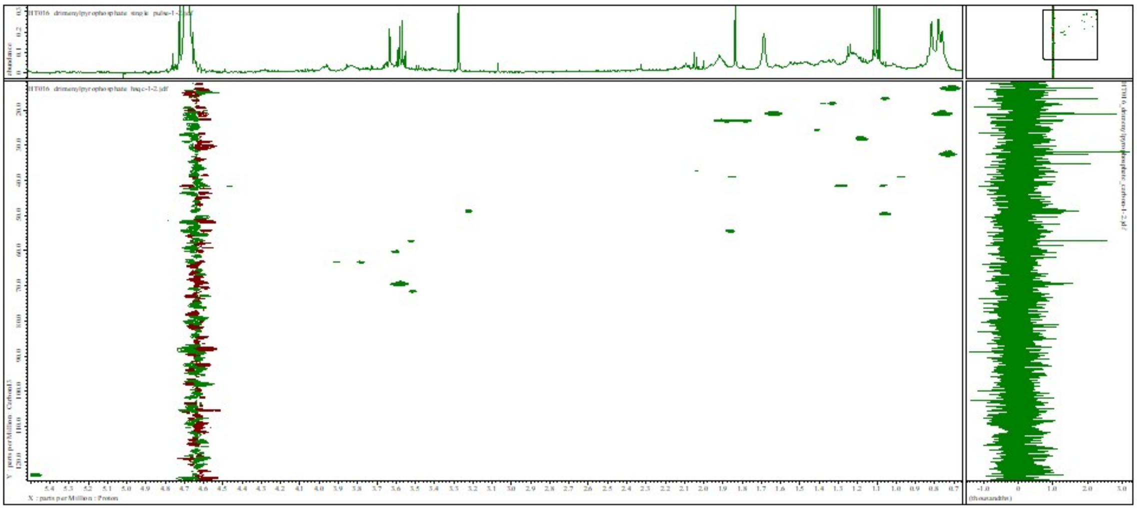

Fig. S11 NMR spectra of **3** in D_2O. (a) ^{1}H- (500 MHz), (b) ^{13}C- (125 MHz), (c) ^{31}P- (202.4 MHz), and (d) HSQC

(a)

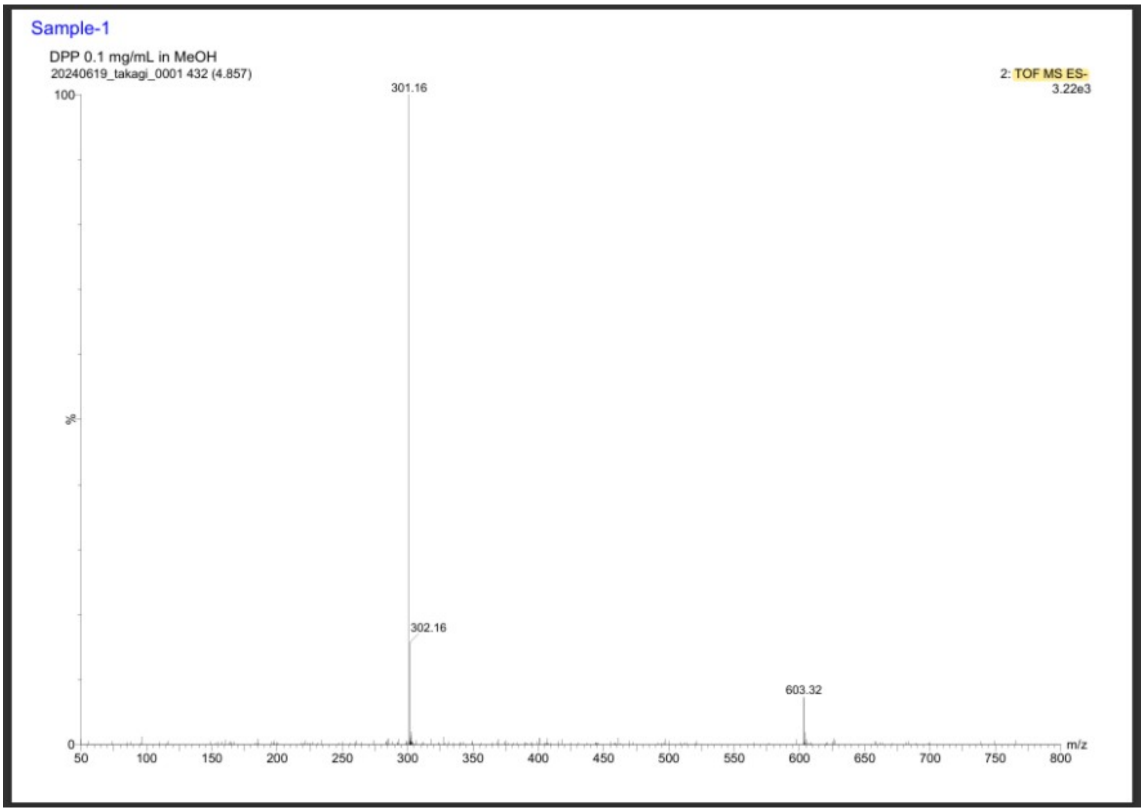

(b)

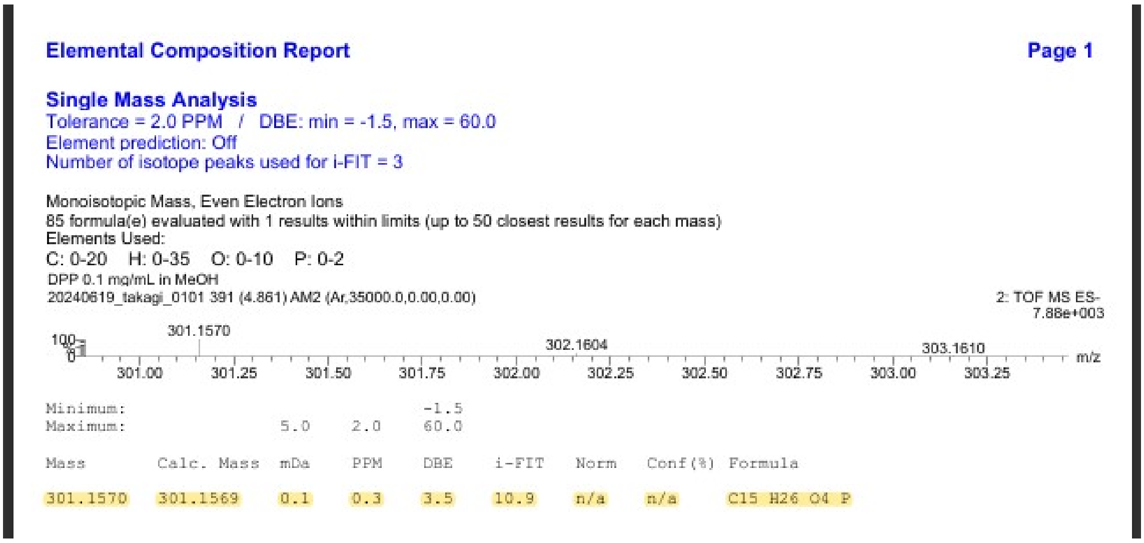

Elemental Composition Report Page 1

Single Mass Analysis
Tolerance = 2.0 PPM / DBE: min = -1.5, max = 60.0
Element prediction: Off
Number of isotope peaks used for i-FIT = 3

Monoisotopic Mass, Even Electron Ions
85 formula(e) evaluated with 1 results within limits (up to 50 closest results for each mass)
Elements Used:
C: 0-20 H: 0-35 O: 0-10 P: 0-2
DPP 0.1 mg/mL in MeOH
20240619_takagi_0101 391 (4.861) AM2 (Ar,35000.0,0.00,0.00)

Minimum:				-1.5				
Maximum:		5.0	2.0	60.0				
Mass	Calc. Mass	mDa	PPM	DBE	i-FIT	Norm	Conf(%)	Formula
301.1570	301.1569	0.1	0.3	3.5	10.9	n/a	n/a	C15 H26 O4 P

Fig. S12 High-resolution LC/MS analysis of purified **3**.

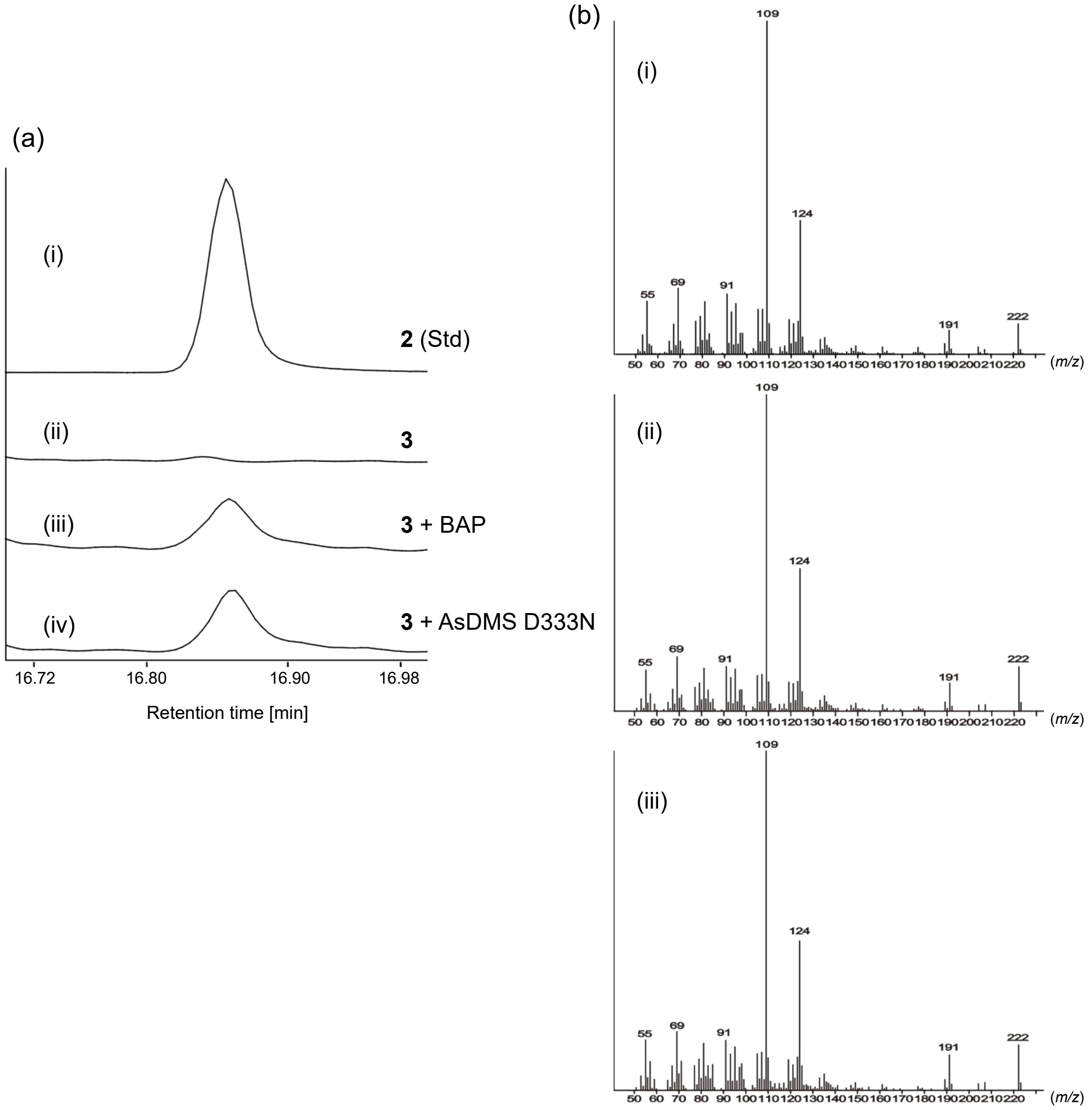

Fig. S13 Enzymatic conversion of **3** to **2**. (a) GC/MS analysis of the dephosphorylation reaction of **3**. Commercially available **2** (i) and purified **3** (ii) were used as the standard. The dephosphorylation reaction was carried out using either alkaline phosphatase (BAP) (iii) or the AsDMS D333N variant (iv). Each sample was analyzed by GC/MS. (b) Mass fragmentation profiles of standard **2** (i), BAP-treated **3** (ii), and **3** treated with the AsDMS D333N variant (iii).

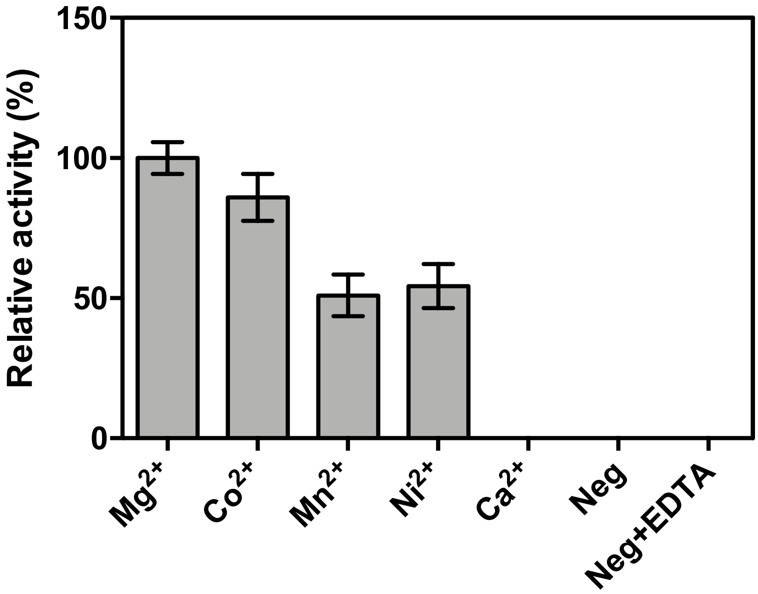

Fig. S14 Divalent metal dependency of AsDMS for **2** production. The enzyme reactions were performed in the presence of **1** substrate and divalent metal ions ($MgCl_2$, $CoCl_2$, $MnCl_2$, $NiCl_2$, or $CaCl_2$). The reactions in the absence of metal ions without (Neg) or with the inclusion of EDTA (Neg + EDTA) were used as negative controls. The catalytic activity of AsDMS in the presence of $MgCl_2$ was set as 100%. Data are the means ± SD of three independent experiments performed with the same preparation of purified AsDMS protein.

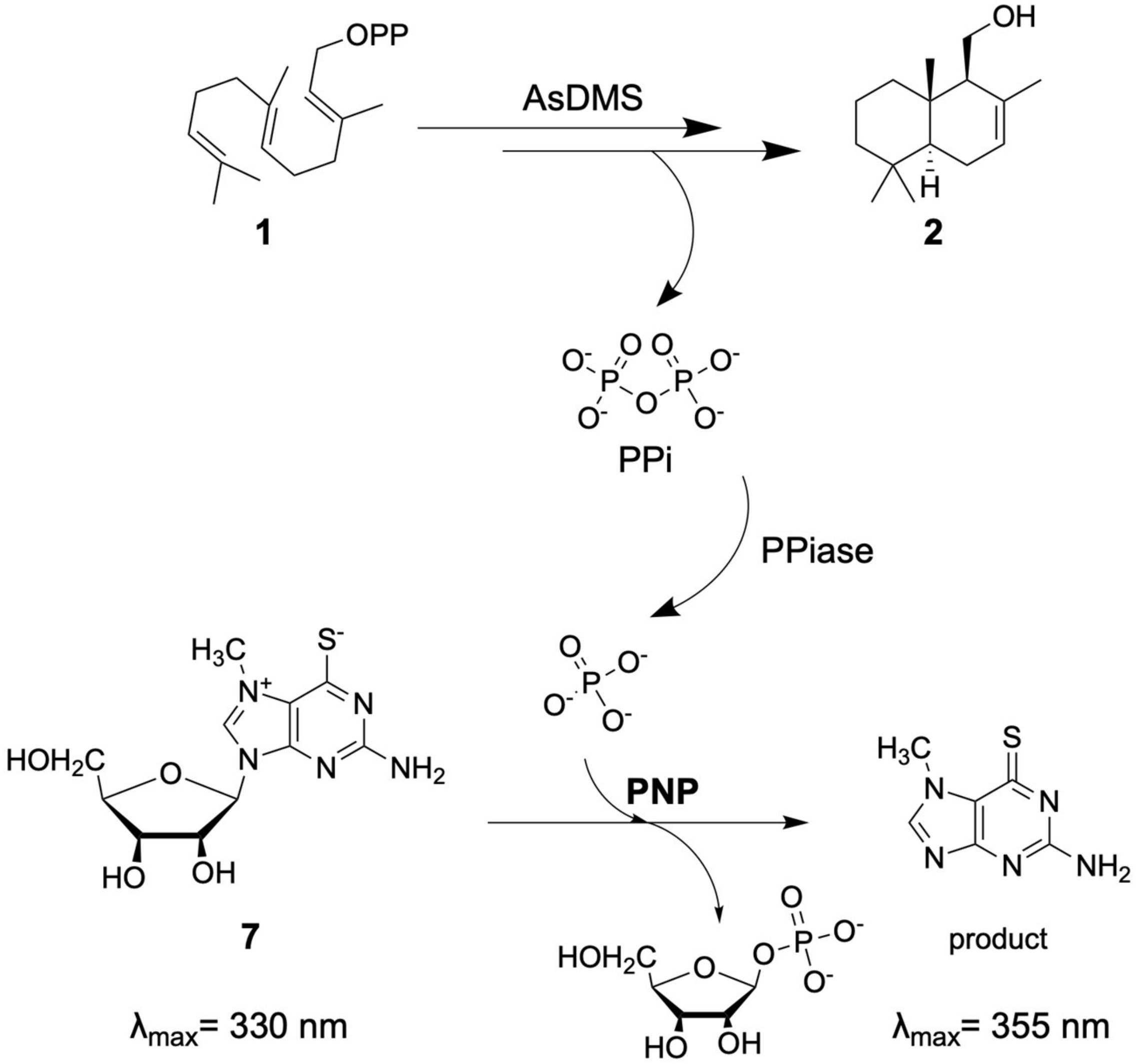

Fig. S15 The MESG assay system detects only inorganic phosphate (Pi) through the conversion of **7** into the MESG product. The MESG product is measured at 360 nm using a spectrophotometer. Given the possibility of pyrophosphate (PPi) production in reactions catalyzed by AsDMS, inorganic pyrophosphatase (PPiase), which converts PPi into Pi, was added to the reaction system.

(a)

		A	B	C	D	E	F	G	H	I	J	K	L	M	N	O
Enzymes	1 µM AsDMS WT	-	-	-	-	-	-	+	+	+	+	+	+	-	-	-
	1 µM AsDMS D333N	-	-	-	-	-	-	-	-	-	-	-	-	+	-	-
	1 µM AsDMS D43A	-	-	-	-	-	-	-	-	-	-	-	-	-	+	+
	2.0 U/mL PPiase	-	-	-	+	+	+	-	-	-	+	+	+	+	+	-
	5.0 U/mL PNP	-	+	+	+	+	+	+	+	+	+	+	+	+	+	+
Substrates	0.1 mM **1**	-	-	-	+	-	-	+	-	-	+	-	-	+	+	+
	0.1 mM **3**	-	-	-	-	+	-	-	+	-	-	+	-	-	-	-
	0.1 mM PPi	-	-	+	-	-	+	-	-	+	-	-	+	-	-	-
	80 µM $MgCl_2$	+	+	+	+	+	+	+	+	+	+	+	+	+	+	+
	0.1 mM **7**	+	+	+	+	+	+	+	+	+	+	+	+	+	+	+
Detection of inorganic phosphate		-	-	-	-	-	+	+	+	-	+	+	-	-	-	-

(b)

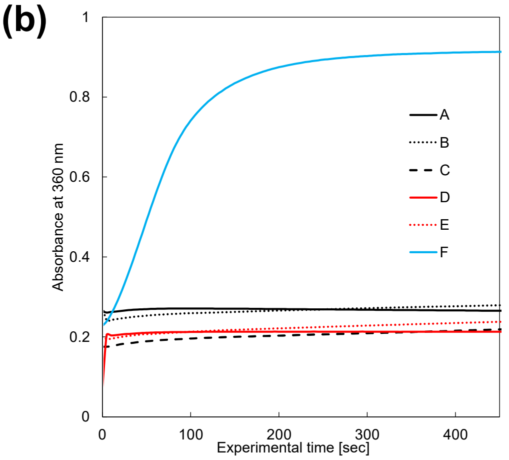

(c)

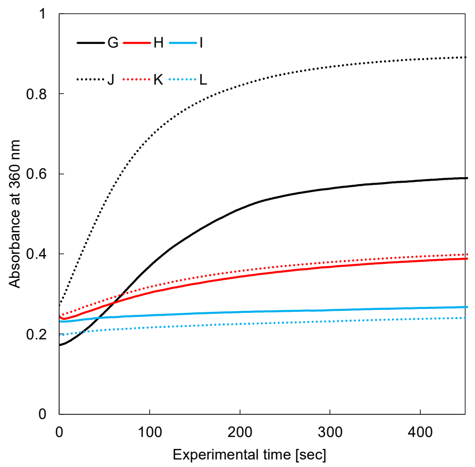

(d)

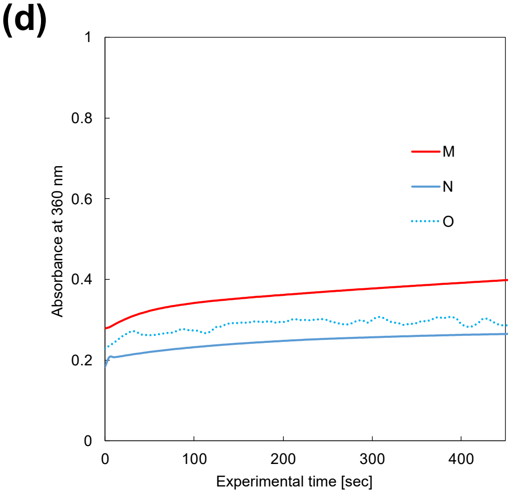

Fig. S16 Detection of AsDMS activity through Pi production using the MESG assay. The composition of the reaction mixture (A–O) is summarized in **(a)**. Raw data from the MESG assay are shown in **(b–d)**.

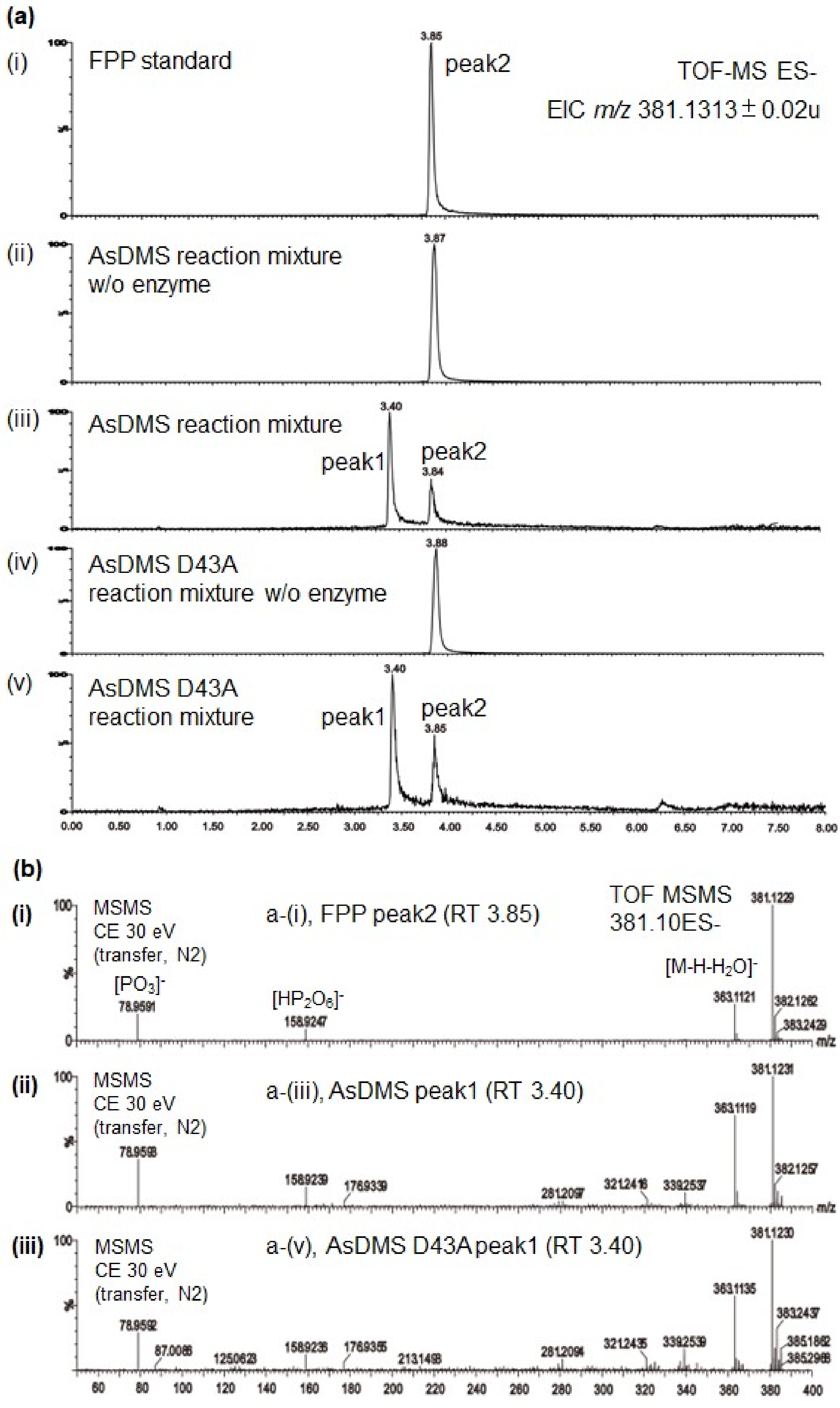

(a)
(i)
FPP standard
peak2
TOF-MS ES-
EIC m/z 381.1313 ± 0.02u
(ii)
AsDMS reaction mixture
w/o enzyme
(iii)
AsDMS reaction mixture
peak1
peak2
(iv)
AsDMS D43A
reaction mixture w/o enzyme
(v)
AsDMS D43A
reaction mixture
peak1
peak2
(b)
(i)
MSMS
CE 30 eV
(transfer, N2)
a-(i), FPP peak2 (RT 3.85)
TOF MSMS
381.10ES-
[PO3]-
[HP2O6]-
[M-H-H2O]-
(ii)
MSMS
CE 30 eV
(transfer, N2)
a-(iii), AsDMS peak1 (RT 3.40)
(iii)
MSMS
CE 30 eV
(transfer, N2)
a-(v), AsDMS D43A peak1 (RT 3.40)

(c)

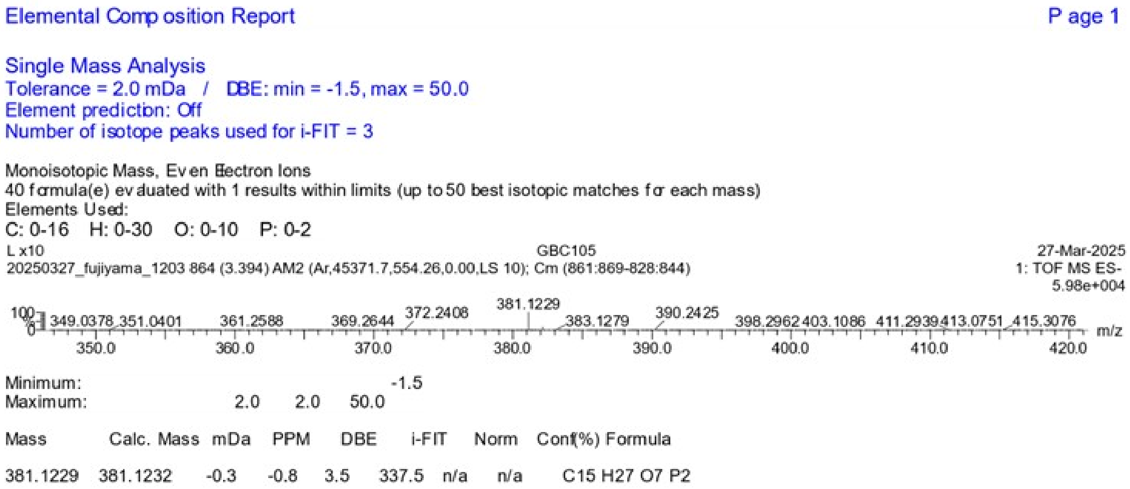

Elemental Composition Report   Page 1

Single Mass Analysis
Tolerance = 2.0 mDa / DBE: min = -1.5, max = 50.0
Element prediction: Off
Number of isotope peaks used for i-FIT = 3

Monoisotopic Mass, Even Electron Ions
40 formula(e) evaluated with 1 results within limits (up to 50 best isotopic matches for each mass)
Elements Used:
C: 0-16 H: 0-30 O: 0-10 P: 0-2

L x10   GBC105   27-Mar-2025
20250327_fujiyama_1203 864 (3.394) AM2 (Ar,45371.7,554.26,0.00,LS 10); Cm (861:869-828:844)   1: TOF MS ES-
5.98e+004

Minimum:				-1.5			
Maximum:		2.0	2.0	50.0			
Mass	Calc. Mass	mDa	PPM	DBE	i-FIT	Norm	Conf(%) Formula
381.1229	381.1232	-0.3	-0.8	3.5	337.5	n/a	n/a C15 H27 O7 P2

Fig. S17 HR-ESI MS analysis of an AsDMS reaction intermediate. (a) EIC chromatogram, (b) HR-TOF-MSMS spectrum, (c) elemental composition report of the AsDMS reaction product corresponding to peak 1. The FPP solution (0.1 mM) was used as a standard (i). Wild-type AsDMS assay was performed in 100 mM Tris-HCl (pH 7.5) containing 0.1 mM FPP and 1mM EDTA, in the absence (ii) and presence (iii) of 1 µM purified AsDMS at 30°C for 1 h. The AsDMS variant assay was performed in 100 mM Tris-HCl (pH 7.5) containing 0.1 mM FPP, in the absence (iv) and presence (v) of 1 µM purified AsDMS D43A variant at 30°C for 1 h. HR-ESI-MS was measured on Waters UPLC-Cyclic IMS system. The following analytical condition was used: column; Waters UPLC Premier C18 2.1 mm x 100 mm (1.7 µm), mobile phase; acetonitrile/10 mM ammonium formate linear gradient, acetonitrile 5% (0.5 min), 5-95% (5.5 min), 95% (2 min), flow rate; 0.3 mL/min.

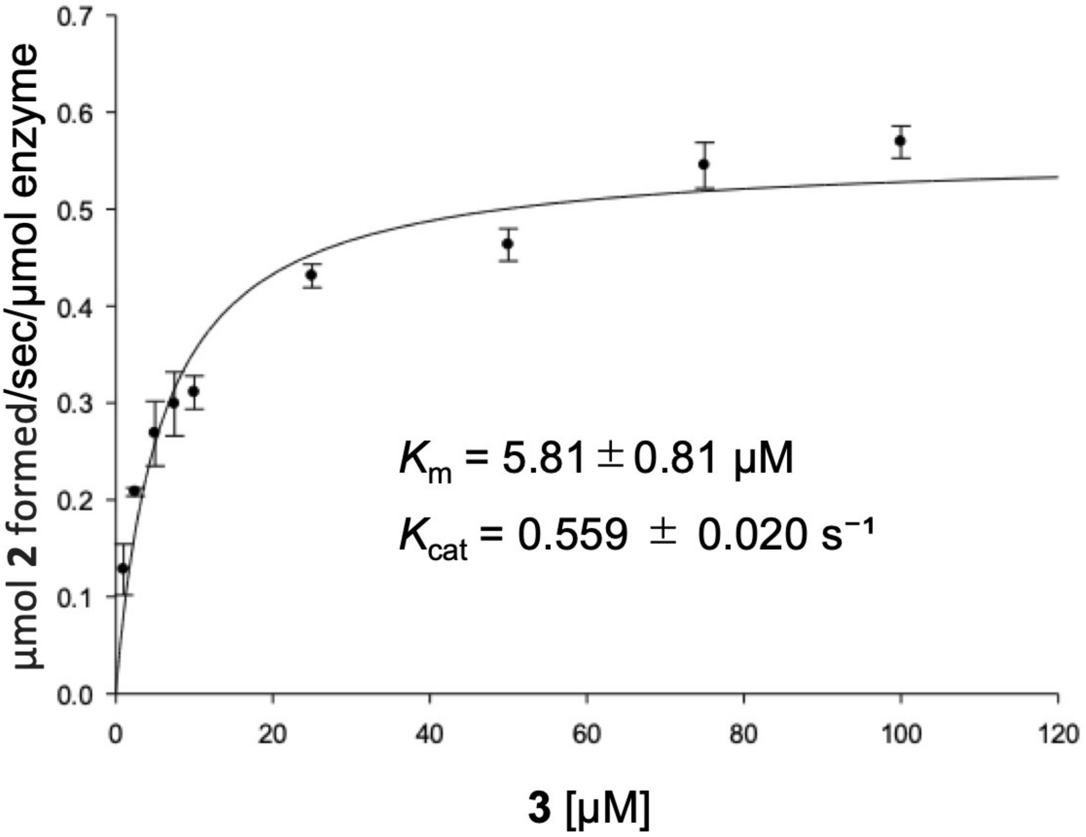

Fig. S18 Kinetic analysis of the AsDMS HAD domain using **3**. The bars represent the standard deviation from three independent experiments. Kinetic constants were calculated by nonlinear regression fitting to the Michaelis-Menten equation.

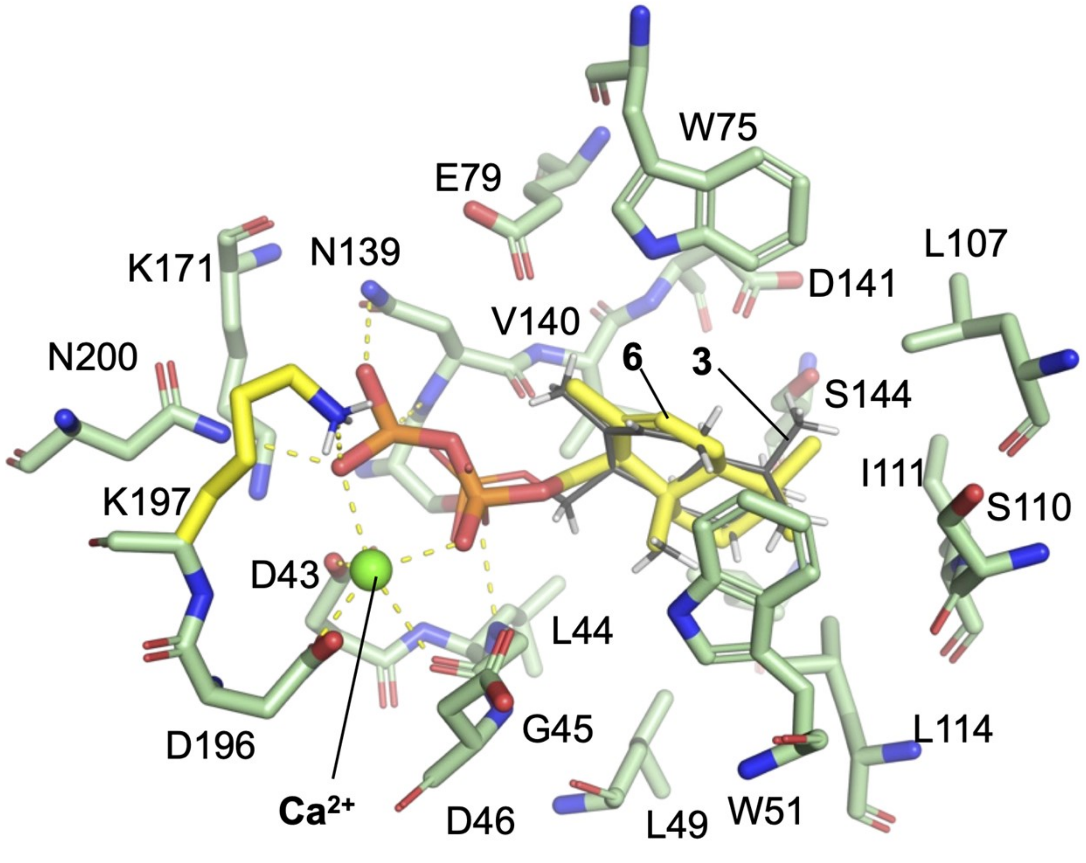

Fig. S19 Docking simulation of the compound **6** in the HAD domain of AsDMS. The yellow dashed lines indicate hydrogen bonds with **6** (within 3.5 Å). The yellow stick models, K197 and compound **6**, were calculated parts. Green stick models and the thin line model of **3** were the crystal structure of **3**-bound AsDMS.

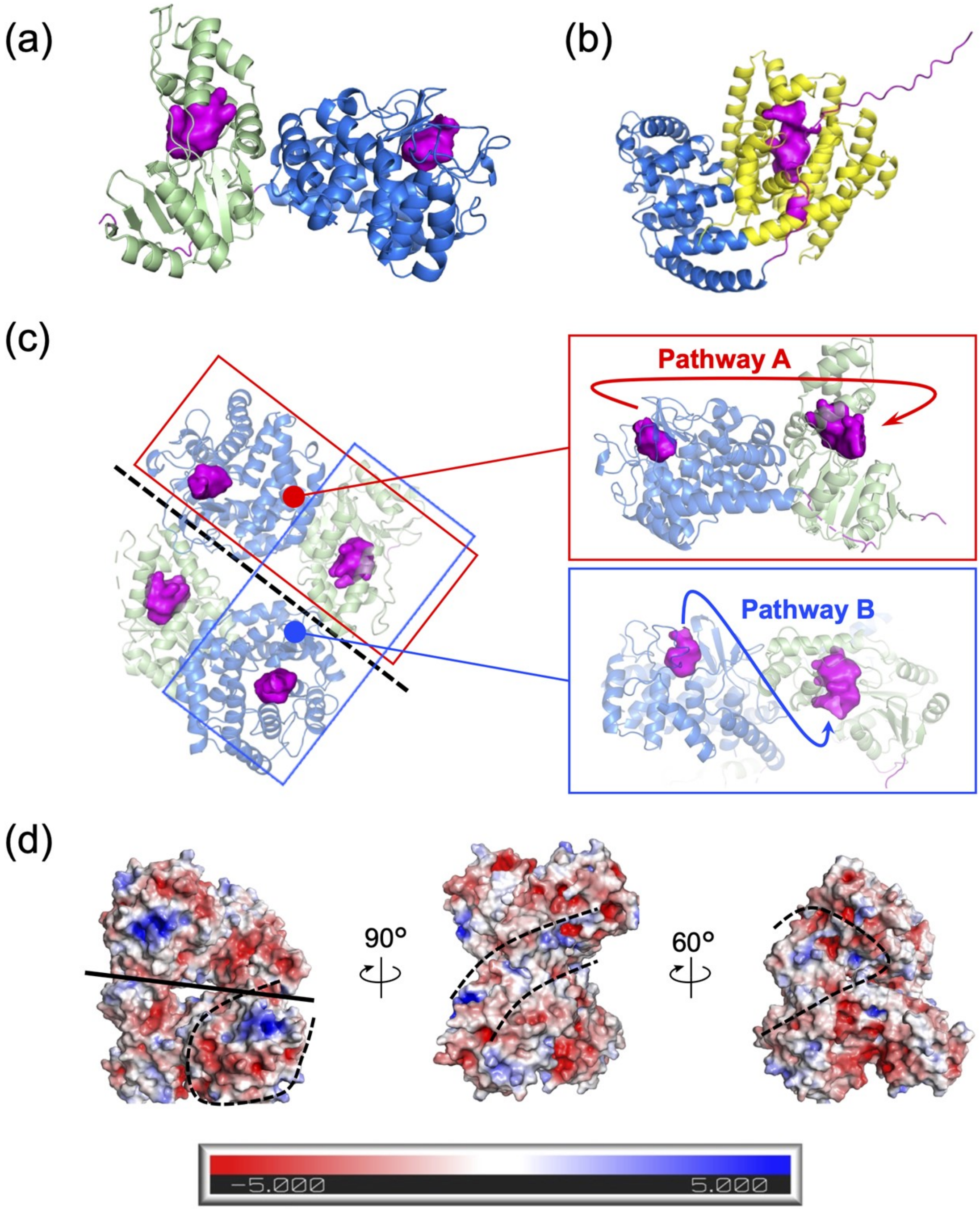

Fig. S20 Putative substrate channeling system of AsDMS. (a) Crystal structure of AsDMS. (b) AlphaFold2-predicted structure of VoDMS.[27] Each substrate-binding site is colored magenta with the surface model. (c) Conceivable intermediate transfer pathways. The dashed line indicates the dimer interface of AsDMS. (d) Visualization of the electrostatic potential at the molecular surface of AsDMS. The solid line indicates the dimer interface of AsDMS. The areas surrounded by dashed lines are positively charged patches of AsDMS.

Scheme S1 Scheme of the overall AsDMS reaction.

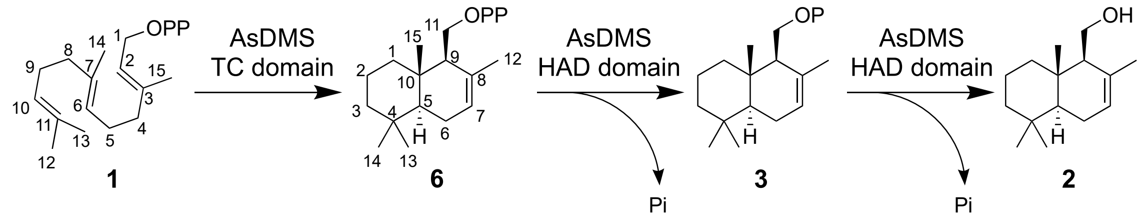

Scheme S2 Resonance forms of the cyclized intermediate in the TCβ domain

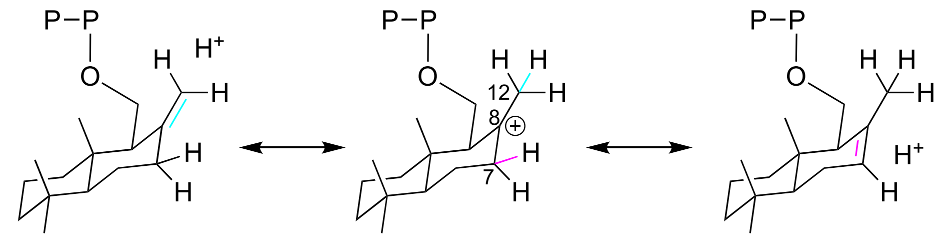

Supporting References

1 N. N. Q. Vo, Y. Nomura, K. Kinugasa, H. Takagi and S. Takahashi, *ACS Chem. Biol.*, 2022, **17**, 1226–1238.

2 Y. Okegawa and K. Motohashi, *Biochem. Biophys.s Rep.*, 2015, **4**, 148–151.

3 Y. Okegawa and K. Motohashi, *Anal. Biochem.*, 2015, **486**, 51–53.

4 W. Kabsch, *Acta Crystallogr. D Biol. Crystallogr.*, 2014, **70**, 2204–2216.

5 P. R. Evans and G. N. Murshudov, *Acta Crystallogr. D Biol. Crystallogr.*, 2013, **69**, 1204–1214.

6 A. J. McCoy, R. W. Grosse-Kunstleve, P. D. Adams, M. D. Winn, L. C. Storoni and R. J. Read, *J. Appl. Crystallogr.*, 2007, **40**, 658–674.

7 M. Mirdita, K. Schütze, Y. Moriwaki, L. Heo, S. Ovchinnikov and M. Steinegger, *Nat. Methods*, 2022, **19**, 679–682.

8 P. Emsley, B. Lohkamp, W. G. Scott and K. Cowtan, *Acta Crystallogr. D Biol. Crystallogr.*, 2010, **66**, 486–501.

9 G. N. Murshudov, P. Skubák, A. A. Lebedev, N. S. Pannu, R. A. Steiner, R. A. Nicholls, M. D. Winn, F. Long and A. A. Vagin, *Acta Crystallogr. D Biol. Crystallogr.*, 2011, **67**, 355–367.

10 P. V. Afonine, R. W. Grosse-Kunstleve, N. Echols, J. J. Headd, N. W. Moriarty, M. Mustyakimov, T. C. Terwilliger, A. Urzhumtsev, P. H. Zwart and P. D. Adams, *Acta Crystallogr. D Biol. Crystallogr.*, 2012, **68**, 352–367.

11 C. J. Williams, J. J. Headd, N. W. Moriarty, M. G. Prisant, L. L. Videau, L. N. Deis, V. Verma, D. A. Keedy, B. J. Hintze, V. B. Chen, S. Jain, S. M. Lewis, W. B. Arendall, J. Snoeyink, P. D. Adams, S. C. Lovell, J. S. Richardson and D. C. Richardson, *Protein Sci.*, 2018, **27**, 293–315.

12 D. Liebschner, P. V. Afonine, N. W. Moriarty, B. K. Poon, O. V. Sobolev, T. C. Terwilliger and P. D. Adams, *Acta Crystallogr. D Biol. Crystallogr.*, 2017, **73**, 148–157.

13 Schrödinger, LLC, ColabFold https://www.nature.com/articles/s41592-022-01488-1 2015.

14 E. Krissinel, *Bioinform.*, 2007, **23**, 717–723.

15 E. Krissinel and K. Henrick, *Acta Crystallogr. D Biol. Crystallogr.*, 2004, **60**, 2256–2268.

16 D. Molodenskiy, E. Shirshin, T. Tikhonova, A. Gruzinov, G. Peters and F. Spinozzi, *Phys. Chem. Chem. Phys.*, 2017, **19**, 17143–17155.

17 M. R. Webb, *Proc. Natl. Acad. Sci. U.S.A.*, 1992, **89**, 4884–4887.

18 X. Pan, W. Du, X. Zhang, X. Lin, F.-R. Li, Q. Yang, H. Wang, J. D. Rudolf, B. Zhang and L.-B. Dong, *J. Am. Chem. Soc.*, 2022, **144**, 22067–22074.

19 G. M. Morris, R. Huey, W. Lindstrom, M. F. Sanner, R. K. Belew, D. S. Goodsell and A. J. Olson, *J. Comput. Chem.*, 2009, **30**, 2785–2791.

20 O. Trott and A. J. Olson, *J. Comput. Chem.*, 2010, **31**, 455–461.

21 D. W. A. Buchan and D. T. Jones, *Nucleic Acids Res.*, 2019, **47**, W402–W407.

22 M. S. Klausen, M. C. Jespersen, H. Nielsen, K. K. Jensen, V. I. Jurtz, C. K. Sønderby, M. O. A. Sommer, O. Winther, M. Nielsen, B. Petersen and P. Marcatili, *Proteins*, 2019, **87**, 520–527.

23 L. Slabinski, L. Jaroszewski, L. Rychlewski, I. A. Wilson, S. A. Lesley and A. Godzik, *Bioinform.*, 2007, **23**, 3403–3405.

24 P. Moosmann, F. Ecker, S. Leopold-Messer, J. K. B. Cahn, C. L. Dieterich, M. Groll and J. Piel, *Nat. Chem.*, 2020, **12**, 968–972.

25 T. Chen, C. Chen, C. Lee, R. Huang, K. Chen, Y. Lu, S. Liang, M. Pham, Y. K. Rao, S. Wu, R. Chein and H. Lin, *Angew. Chem. Int. Ed.*, 2023, **62**, e202215566.

26 Y. Shinohara, S. Takahashi, H. Osada and Y. Koyama, *Sci. Rep.*, 2016, **6**, 32865.

27 M. Kwon, S. A. Cochrane, J. C. Vederas and D.-K. Ro, *FEBS Lett.*, 2014, **588**, 4597–4603.

28 X. Robert and P. Gouet, *Nucleic Acids Res.*, 2014, **42**, W320–W324.
